# Foamy monocytes and atherogenesis in mice with combined hyperlipidemia and effects of antisense knockdown of apoCIII

**DOI:** 10.1016/j.jlr.2025.100763

**Published:** 2025-02-21

**Authors:** Xueying Peng, Zeqin Lian, Veronica O'Brien, Jing Xiao, Benjamin A. Litchfield, Xiao-Yuan Dai Perrard, Lu Xu, Jing Ni, Aparna Mukherjee, Timothy Simmons, Henry Dong, Adam E. Mullick, Rosanne Crooke, Henry J. Pownall, Scott I. Simon, Christie M. Ballantyne, Huaizhu Wu

**Affiliations:** 1 Department of Clinical Pharmacy, Key Laboratory of Clinical Cancer Pharmacology and Toxicology Research of Zhejiang Province, Affiliated Hangzhou First People's Hospital, Westlake University School of Medicine, Hangzhou, Zhejiang, PR China; 2 Department of Medicine, Baylor College of Medicine, Houston, TX, USA; 3 Department of Pediatrics, Children's Hospital of Pittsburgh UPMC, University of Pittsburgh School of Medicine, Pittsburgh, PA, USA; 4 Ionis Pharmaceuticals, Carlsbad, CA, USA; 5 Center for Bioenergetics, Houston Methodist Research Institute, Houston, TX, USA; 6 Department of Biomedical Engineering, University of California, Davis, CA, USA; 7 Center for Cardiometabolic Disease Prevention, Baylor College of Medicine, Houston, TX, USA

**Keywords:** atherosclerosis, cholesterol, foam cells, inflammation, lipoproteins, monocytes, triglyceride

## Abstract

Hypertriglyceridemia (HTG), particularly in combined hyperlipidemia, increases risk for atherosclerotic cardiovascular disease, but the underlying mechanisms remain incompletely understood. We sought to determine contributions of circulating monocytes to atherosclerosis associated with HTG in combined hyperlipidemia, created by transgenic expression of human apoCIII in *Ldlr*^−/−^ mice (*Ldlr*^−/−^ApoCIIItg) fed Western high-fat diet (WD). Tissue culture with THP-1 and primary human monocytes was used to examine effects of triglyceride (TG)-rich lipoproteins on monocytes. *Ldlr*^−/−^ApoCIIItg mice were also treated with apoCIII antisense oligonucleotide (ASO) and examined for foamy monocytes and atherosclerosis. Compared to *Ldlr*^−/−^ mice, *Ldlr*^−/−^ApoCIIItg mice fed WD had early and persistent increases in lipid accumulation within monocytes and enhanced atherosclerosis. *Ldlr*^−/−^ApoCIIItg mice versus *Ldlr*^−/−^ mice had higher levels of CD11c, CD36, and cytokines in foamy monocytes, with increases in foamy monocyte adhesion to vascular cell adhesion molecule-1 and oxidized LDL uptake. Monocytes took up TG-rich lipoprotein in vivo and in vitro and changed phenotypes. Foamy monocytes infiltrated into atherosclerotic lesions, and specific and sustained depletion of CD11c^+^ (foamy) monocytes profoundly reduced atherosclerosis in *Ldlr*^−/−^ApoCIIItg mice on WD. Treatment with apoCIII ASO lowered plasma TG and cholesterol levels, improved foamy monocyte phenotypes, and reduced atherosclerosis in *Ldlr*^−/−^ApoCIIItg mice. In conclusion, HTG in combined hyperlipidemia accelerates atherosclerosis, in part, by increasing foamy monocyte formation and infiltration into atherosclerotic plaques. Treatment with apoCIII ASO is a potential new therapy for improving monocyte phenotypes and reducing atherosclerosis in combined hyperlipidemia.

Atherosclerosis underlies most ischemic cardiovascular disease, the major cause of death in the United States and worldwide. As a key risk factor for atherosclerosis, hypercholesterolemia has been the major therapeutic target for atherosclerotic cardiovascular disease (ASCVD) prevention (1). From recent genetic and epidemiologic studies, hypertriglyceridemia (HTG), particularly in combined hyperlipidemia (with elevated non-HDL-cholesterol), is also causally linked to ASCVD risk (2, 3, 4). However, how HTG contributes to atherosclerosis remains incompletely understood.

Atherosclerosis is an inflammatory process characterized mainly by lipid-laden macrophage (foam cell) accumulation within arterial walls (5, 6). Infiltration of blood monocytes into arterial walls, with differentiation into macrophages, is a key step in atherogenesis (6, 7, 8, 9, 10, 11, 12). However, compared to studies of tissue macrophage-lipoprotein interactions and foam cell formation in arterial walls, studies of circulating monocyte-lipoprotein interactions and their effects on monocyte phenotypes and atherogenesis are limited. We previously reported that in mice with hypercholesterolemia, circulating monocytes take up cholesteryl ester-rich lipoproteins and become lipid-laden “foamy monocytes,” which change phenotypes, migrate into arterial walls, and contribute to atherosclerosis (6, 8, 13). Similarly, in human familial hypercholesterolemia, circulating monocytes contain increased levels of intracellular lipids and display proinflammatory phenotypes, which may contribute to premature ASCVD (7, 14).

HTG in humans or mice is also associated with increased monocyte lipid accumulation and monocyte phenotypic changes (15, 16, 17). In fact, HTG induced by a single high-fat meal in human subjects can increase monocyte lipid accumulation and change monocyte phenotypes (15, 18, 19, 20). On the other hand, treatment with n-3 fatty acids, including eicosapentaenoic acid, reduces plasma triglyceride (TG) levels, decreases monocyte lipid accumulation, and improves monocyte phenotypes in humans with HTG (21). Of note, the Reduction of Cardiovascular Events with Icosapent Ethyl-Intervention Trial showed that eicosapentaenoic acid treatment lowers the risk for ASCVD in subjects with HTG (22). Nevertheless, how monocytes respond to HTG in combined hyperlipidemia and subsequently contribute to atherosclerosis remains to be determined.

Thus, using mouse models with HTG in combined hyperlipidemia induced by transgenic expression of human apoCIII (ApoCIIItg) in *Ldlr*^−/−^ mice and tissue culture, we tested a hypothesis that HTG in combined hyperlipidemia compared to hypercholesterolemia alone enhances monocyte lipid accumulation and phenotypic changes, which facilitate monocyte infiltration into atherosclerotic plaques and increase foam cell formation, thereby accelerating atherogenesis.

## Materials and methods

Please see the extended Materials and Methods section in the Supplemental Data.

### Animals and diet

Male *Ldlr*^−/−^ApoCIIItg (*Ldlr*^−/−^ApoCIII^+^) mice and *Ldlr*^−/−^ controls (*Ldlr*^−/−^ApoCIII^–^ littermates) generated by crossing *Ldlr*^−/−^ (strain #002207; The Jackson Laboratory, Bar Harbor, ME) with ApoCIIItg mice (strain #006907; The Jackson Laboratory) were used for animal studies, which were approved by the Institutional Animal Care and Use Committee of Baylor College of Medicine. Mice were fed a normal laboratory diet (ND; 5% fat [w/w]; Rodent Diet 5010, LabDiet, St. Louis, MO) and, at age 8 weeks, switched to Western high-fat diet (WD) (21% milkfat [w/w], 0.2% cholesterol; Dyet 112734, Dyets, Inc, Bethlehem, PA) and maintained on WD for 6-13 weeks. Blood was collected by facial vein puncture or by cardiac puncture when mice were euthanized via inhalation of 5% or greater isoflurane (USP, NDC: 11695-6777-2; Covetrus, Portland, ME), which was continued until 1 min after breathing stopped, followed by cervical dislocation. Mouse aorta and heart were collected and fixed in 4% paraformaldehyde in PBS or embedded in optimal cutting temperature compound (Sakura Finetek, Torrance, CA). In addition, insulin tolerance test was performed in a cohort of mice fed WD (for 6 weeks) after fasting for 6 h as described previously (23).

### Human subjects and TG-rich lipoprotein isolation

Human subjects with metabolic syndrome were recruited. Blood was collected at 5 h after intake of a high-fat meal (containing ∼900 kcal, ∼51% of which was from fat and ∼23% from saturated fat) (15). TG-rich lipoproteins (TGRL) were isolated from postprandial plasma by ultracentrifugation with precaution to avoid endotoxin contamination and, in some cases, labeled with DiI (Sigma-Aldrich, St. Louis, MO) (8, 13). The study was performed in compliance with the principles of the Declaration of Helsinki under the protocol #H-21418, which was approved by the Institutional Review Board of Baylor College of Medicine, and all participants provided written informed consent prior to participation.

### Treatment of mice with antisense oligonucleotide against apoCIII

A GalNac-conjugated antisense oligonucleotide (ASO) against human apoCIII and a GalNac-conjugated control oligonucleotide (CO) were provided by Ionis Pharmaceuticals (Carlsbad, CA) (24). Male *Ldlr*^−/−^ApoCIIItg mice were fed WD for 1 week, then mice with corresponding TG levels were randomized into CO and ASO treatment groups, which received weekly subcutaneous injection of 10 mg/kg CO and ASO, respectively. An *Ldlr*^−/−^ control group receiving weekly subcutaneous injection of CO was also included. Blood was collected weekly or biweekly via facial vein. Plasma TG and cholesterol were determined by standard enzymatic assays (Wako Diagnostics, Richmond, VA). Monocyte phenotypes were examined by fluorescence-activated cell sorting (FACS) analysis. After treatment for 12 weeks, mice were euthanized, and aorta and heart were collected for analyses of atherosclerosis. A separate cohort of *Ldlr*^−/−^ApoCIIItg mice fed WD were treated with CO or ASO for 6 weeks, and monocytes were isolated and analyzed for lipid content. In another cohort, *Ldlr*^−/−^ mice fed WD were treated with GalNac-conjugated ASO against mouse apoCIII or CO via weekly subcutaneous injection at 15 mg/kg for 6 weeks, during which plasma levels of TG and cholesterol were monitored. At the end of the treatment period, monocyte lipids were quantified using commercial kits (see below), and plasma apoCIII levels were measured using a mouse apoCIII ELISA kit (Avantor, Inc) following the manufacturer’s instructions.

### Plasma lipoprotein profiles

Plasma lipoprotein profiles were determined by size-exclusion chromatography (Superose HR6 column, GE HealthCare, Chicago, IL) as previously described (13, 25). Cholesterol and TG concentrations in each fraction were determined enzymatically using commercial kits (Wako Diagnostics).

### Antibodies and FACS analysis of circulating monocytes

Monocyte phenotypes were analyzed by FACS using monoclonal antibodies (mAbs) to mouse antigens, including CD115 (PE, AFS98, ThermoFisher Scientific, Waltham, MA; or BV421, AFS98, BioLegend, San Diego, CA), CD11c (PerCP-Cy5.5, N418, ThermoFisher Scientific; or APC or FITC, N418, BioLegend), CD36 (FITC, MF3, Bio-Rad Laboratories, Hercules, CA), CD204 (FITC, 2F8, Bio-Rad Laboratories), Ly-6C (APC, HK1.4, ThermoFisher Scientific), CX3CR1 (FITC, SA011F11, BioLegend), MHCII (I-A/I-E; PE, M5/114, BD Biosciences, San Jose, CA), CD43 (PE, S11, BioLegend), TREML4 (PE, 16E5, BioLegend), XCR1 (PE, ZET, BioLegend), tumor necrosis factor α (TNFα; PE, MP6-XT22, ThermoFisher Scientific), and interleukin-1β (IL-1β; PE, NJTEN3, ThermoFisher Scientific), with appropriate isotype-negative controls. Data were collected on a BD LSRII (BD Biosciences) or Beckman Coulter CytoFLEX (Beckman Coulter, Brea, CA) flow cytometer and analyzed using Kaluza (Beckman Coulter) or FlowJo (Tree Star, Inc, Ashland, OR) software (see Supplemental Data and supplemental Fig. S1) (13, 25).

### Detection of cellular TG and cholesterol in mouse monocytes

TG, free glycerol, total cholesterol, and cholesteryl esters in mouse monocytes were measured using the Triglyceride-Glo assay kit (Promega, Madison, WI) and Cholesterol/Cholesterol Ester-Glo assay kit (Promega) according to the manufacturer’s instructions (26). Briefly, monocytes were isolated from mouse blood by enrichment of white blood cells using RosetteSep™ DM-M (StemCell Technologies, Vancouver, BC) followed by negative selection using PE-conjugated mAbs against mouse CD3 (17A2, BioLegend), CD19 (1D3, BioLegend), and Ly-6G (1A8, BioLegend) and separation using mouse anti-PE-nanobeads (BioLegend). Cells (25,000 in 50 μl lysis solution) were added to each well. Glycerol and cholesterol content was measured in cell lysates and normalized to the cell number.

### Mouse monocyte uptake of TGRL and oxidized LDL

Mouse TGRL (mTGRL) were isolated by ultracentrifugation from EDTA plasma of *Ldlr*^−/−^ApoCIIItg mice fed WD and were labeled with DiI (8). To examine mouse monocyte uptake of TGRL or oxidized LDL (OxLDL) ex vivo, heparinized blood collected from mice was washed with PBS to remove endogenous lipoproteins and incubated with DiI-mTGRL (at 200 mg/dl TG) or DiI-OxLDL (0.05 mg protein/ml; Kalen Biomedical, Montgomery Village, MD) in RPMI-1640 medium at 37°C for 3 h, with an anti-mouse CD36 mAb (4 μg/ml) (JC63.1; Abcam, Cambridge, UK) or with an isotype-negative control. After red blood cells were lysed with RBC lysis buffer (BioLegend), the samples were stained for CD204 and CD11c. Monocyte uptake of DiI-TGRL or DiI-OxLDL was evaluated by FACS analysis (8, 13). In addition, an aliquot of the stained samples was also fixed with RBC Lysis/Fixation Solution (BioLegend), stained with 4',6-diamidino-2-phenylindole (DAPI) for cell nuclei, and visualized by a Zeiss LSM780 confocal microscope (Zeiss Microscopy, Oberkochen, Germany).

To examine monocyte uptake of TGRL in vivo, DiI-mTGRL (equivalent to 100 mg/dl TG) were injected into mice at 0.3 ml/mouse via tail vein. After 24 h, blood was collected and monocyte uptake of TGRL was analyzed by FACS after staining for CD204 and CD11c (6, 8).

### Monocyte adhesion assay

The chip microfluidic assembly and monocyte adhesion assay were performed as previously reported (13). Briefly, clean coverslips that were coated with mouse recombinant vascular cell adhesion molecule-1 (VCAM-1)-Fc chimera and E-selectin-Fc chimera (R&D Systems, Minneapolis, MN) were assembled with a 4-channel polydimethylsiloxane device and kept on a 37°C surface. Heparinized blood from mice was stained with PE-anti-mouse CD115 and FITC-anti-mouse CD11c (N418; BioLegend) at room temperature for 20 min and then diluted 1:3 in PBS containing calcium and magnesium. Diluted stained blood (60 μl) was introduced into the channel at a flow rate that produced a shear stress of 2 dyn/cm^2^ at fluid-glass interface and perfused for 5 min followed by fixation and mounting in DAPI medium. The number of adherent monocytes was counted and normalized by total infused monocyte number.

### In vivo monocyte trafficking

To examine monocyte trafficking in *Ldlr*^−/−^ApoCIIItg mice, two protocols were used. In protocol 1, a bolus of fluorescent microbeads (Fluoresbrite® YG Carboxylate Microspheres; Polysciences, Inc, Brentwood, TN) was injected intravenously into *Ldlr*^−/−^ApoCIIItg mice fed WD (6 weeks) to specifically label CD11c^+^ (Ly-6C^low^) monocytes (8, 9). In protocol 2, DiI-mTGRL (equivalent to 100 mg/dl TG) were intravenously injected into C57BL/6J WT mice (donors) at 0.3 ml/mouse. After 24 h, blood was drawn from donors. Monocyte uptake of DiI-TGRL was examined (see Results section). Mononuclear cells were isolated using Histopaque (Sigma-Aldrich), and, after being washed, were injected intravenously into recipient *Ldlr*^−/−^ApoCIIItg mice fed WD (12 weeks) at 2.3 × 10^6^/recipient. Twenty-four hours later, the same recipients received another injection of labeled mononuclear cells from WT. At 48 h after microbead injection (protocol 1) or the second injection of mononuclear cells (protocol 2), hearts of *Ldlr*^−/−^ApoCIIItg mice were collected and sectioned. The sections were stained for CD11c (protocol 1) and nuclei (using DAPI, protocols 1 and 2), and images were captured using an EVOS fl Fluorescence Microscope (Life Technologies, Carlsbad, CA) to examine microbead- or DiI-TGRL-labeled monocyte trafficking into lesions.

### Monocyte depletion

CD11c^+^ (mainly CD36^+^ and Ly-6C^low^) monocytes were depleted in mice using low-dose clodrosome (Encapsula NanoScience LLC, Nashville, TN). Mice fed WD received intravenous injection of clodrosome daily (at 0.1 ml/mouse after 1:5 dilution in saline) for 6 weeks (8), during which blood was collected weekly to examine monocytes and plasma lipid levels. After 6 weeks, mice were euthanized. Heart and whole aorta were collected for analyses of atherosclerosis.

### Atherosclerotic lesion analysis

We adhered to the guidelines for experimental atherosclerosis studies as described in the American Heart Association scientific statement (27). Briefly, mouse aortas were harvested and fixed in 4% paraformaldehyde in PBS. After removal of outside fat and connecting tissue, aortas were cut longitudinally and stained with Oil Red O (0.5 g/100 ml 60% 2-propanol; Sigma-Aldrich). Digital images were captured with a Nikon camera (Nikon Instruments, Melville, NY). Atherosclerotic plaque areas that were Oil Red O-positive were quantitated using ImageJ analysis software (National Institutes of Health, Bethesda, MD) (8, 13, 25).

To analyze atherosclerosis in the aortic root, mouse hearts embedded in optimal cutting temperature medium were sectioned using a freezing microtome (Leica CM3050 S; Leica Biosystems, Deer Park, IL) by cutting serial cross sections at 5 μm/section starting from the appearance until the disappearance of the leaflets of the aortic valves. The sections were stained with Oil Red O or with PE-conjugated anti-CD11c mAb (ThermoFisher Scientific) for lesion analyses and also processed for immunohistochemistry staining for human apoCIII using a primary antibody (goat IgG; Abcam) followed by the ImmPRESS HRP Horse Anti-Goat IgG Detection Polymer Kit and an ImmPACT DAB substrate kit (Vector Laboratories, Newark, CA) (6). Images were captured using a microscope coupled with NIS-Elements software (Nikon Eclipse Ci) or an EVOS fl Fluorescence Microscope and analyzed using ImageJ software or Adobe Photoshop by at least two investigators in a blinded manner (8, 13, 25). Imaging parameters and software setup were constant for all photomicrograph acquisitions in an experiment. For each sample, six sections at 200-μm intervals from the aorta root (whole length of aortic valve) were used for quantification of the plaque size. Data were presented as the average of the six sections per sample. The lumen side was selected as the region of interest, which was recognized as the space inside elastic fibers of the aorta. The area, of which the color intensity was higher than the threshold justified by the negative-control section, was selected as the positive-staining region.

### Cell culture

To examine human monocyte uptake of TGRL, THP-1 monocytes (ATCC, Manassas, VA) or blood from healthy subjects (after being washed with PBS to remove endogenous lipoproteins) were incubated with DiI-human TGRL at 200 mg/dl TG in the presence or the absence of 100 μM orlistat (a lipase inhibitor; Sigma-Aldrich) in RPMI-1640 medium for 4 h at 37°C and then analyzed by FACS (after staining for CD14 in human blood) as described previously. THP-1 monocytes were also treated with unlabeled human TGRL (200 mg/dl TG) in the presence or the absence of 100 μM orlistat or, in separate experiments, of 2 units/ml lipoprotein lipase (LPL; Sigma-Aldrich) in RPMI-1640 medium (supplemented with 3% fatty acid-free low-endotoxin bovine serum albumin [Sigma-Aldrich]) for 24–48 h at 37°C. Monocyte lipid accumulation and phenotypic changes were examined by FACS and quantitative RT-PCR.

In separate experiments, THP-1 monocytes were treated with unlabeled TGRL (200 mg/dl TG) for 48 h and then, after being washed with PBS to remove TGRL, incubated with DiI-OxLDL in the presence of a CD36 mAb (4 μg/ml, FA6-152, or an isotype-negative control, StemCell Technologies) for an additional 4 h at 37°C. Monocyte uptake of DiI-OxLDL was examined by FACS.

### RNA isolation and quantitative RT-PCR

Total RNA was isolated from tissues or cells using TRIzol Reagent (ThermoFisher Scientific) and an RNA Miniprep Kit (Zymo Research, Irvine, CA). mRNA levels of target genes were examined by quantitative RT-PCR using predesigned primers and probes with TaqMan Universal PCR Master Mix (ThermoFisher Scientific) and expressed as relative values to 18S rRNA.

### Statistical analysis

Statistical analyses were performed in GraphPad Prism 8.3 or higher (GraphPad Software, San Diego, CA). Scatter dot plots and error bars were presented as mean ± SEM. The Mann-Whitney or Kruskal-Wallis test followed by Dunn's multiple pairwise comparisons was used for comparisons between two groups or ≥3 groups for data that were not normally distributed or did not have equivalence of variance. Otherwise, the unpaired or paired Student’s *t* test or one-way ANOVA followed by Tukey's or Dunnett’s multiple pairwise comparisons test was used for 2-group or ≥3-group comparisons. For comparisons among groups over time or across lipoprotein fractions, two-way ANOVA with repeated measures and assumption of inequivalence of variance followed by Tukey’s or Sidak’s multiple pairwise comparisons test was used. The differences were considered statistically significant at *P* ≤ 0.05.

## Results

### Combined hyperlipidemia and increased atherosclerosis in *Ldlr*^−/−^ApoCIIItg mice

**Figure 1:**
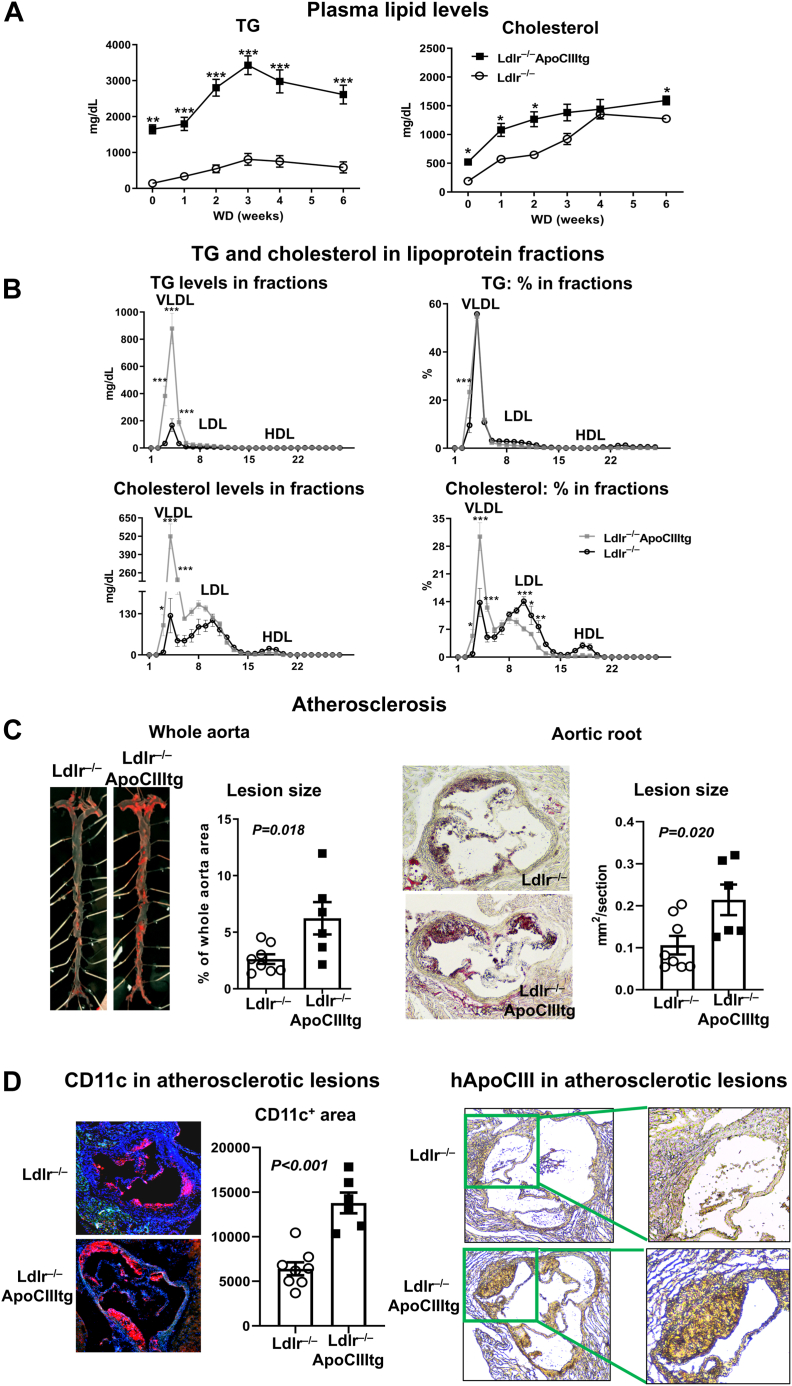
Increased plasma TGs and atherosclerosis in *Ldlr*^−/−^ mice with transgenic expression of human apoCIII (*Ldlr*^−/−^apoCIIItg). A: *Ldlr*^−/−^ApoCIIItg and *Ldlr*^−/−^ mice were fed WD for 6 weeks. Plasma total TG and total cholesterol levels (n = 4–9 samples/group). B: TG and cholesterol distributions in lipoprotein fractions fractionated by size-exclusion chromatography. Data are presented as TG and cholesterol levels and percentage in each lipoprotein fraction (n = 3–4 samples/group). C: Representative photographs and quantitative analysis of en face Oil Red O staining for atherosclerotic lesions in whole aorta and aortic sinus in mice (n = 6–8 mice/group). D: Staining for CD11c and human apoCIII (hApoCIII) in aortic sinus lesions in mice. ∗*P* < 0.05 and ∗∗∗*P* < 0.001 compared to *Ldlr*^−/−^ group.

Based on our research focus on monocyte contributions to atherogenesis, which were previously shown to be greater in the early stage of atherogenesis (12), we chose 6-week WD feeding for most of our studies. Compared to *Ldlr*^−/−^ counterparts, *Ldlr*^−/−^ApoCIIItg mice had similar weight gains, with comparable body weight (supplemental Fig. S2A) and epididymal adipose tissue weight (supplemental Fig. S2B), but improved insulin sensitivity indicated by insulin tolerance test (supplemental Fig. S2C), at 6 weeks on WD. Plasma TG levels were ∼4- to 5-fold higher, and total cholesterol levels were ∼1.5-fold higher in *Ldlr*^−/−^ApoCIIItg mice than in *Ldlr*^−/−^ controls (Fig. 1A), consistent with previous reports indicating HTG and combined hyperlipidemia in *Ldlr*^−/−^ApoCIIItg mice (28, 29). Size-exclusion chromatography analysis showed that *Ldlr*^−/−^ApoCIIItg mice compared to *Ldlr*^−/−^ controls had higher TG levels in VLDL (Fig. 1B). As expected, TG was enriched mainly in VLDL fractions in both *Ldlr*^−/−^ApoCIIItg and *Ldlr*^−/−^ mice, and *Ldlr*^−/−^ApoCIIItg mice had greater TG enrichment in VLDL than did *Ldlr*^−/−^ mice (Fig. 1B). Cholesterol levels were higher or tended to be higher in VLDL and LDL in *Ldlr*^−/−^ApoCIIItg mice than in *Ldlr*^−/−^ controls. *Ldlr*^−/−^ApoCIIItg mice compared to *Ldlr*^−/−^ controls had higher percentage of cholesterol in VLDL but lower percentage of cholesterol in LDL (Fig. 1B).

Compared to *Ldlr*^−/−^ controls, *Ldlr*^−/−^ApoCIIItg mice fed WD for 6 weeks had significant increases in atherosclerotic plaque size in the whole aorta and aortic roots as examined by Oil Red O staining (Fig. 1C), consistent with previous reports showing increased atherosclerosis in *Ldlr*^−/−^ApoCIIItg mice fed WD for 3 months (28, 29). Immunostaining of aortic roots showed that *Ldlr*^−/−^ApoCIIItg mice compared to *Ldlr*^−/−^ controls had greater CD11c^+^ foam cell areas in atherosclerotic lesions (Fig. 1D). By immunostaining, we also observed human apoCIII in atherosclerotic lesions of *Ldlr*^−/−^ApoCIIItg mice (Fig. 1D).

### Changes in monocyte proportions and lipid accumulation in *Ldlr*^−/−^ApoCIIItg mice

**Figure 2:**
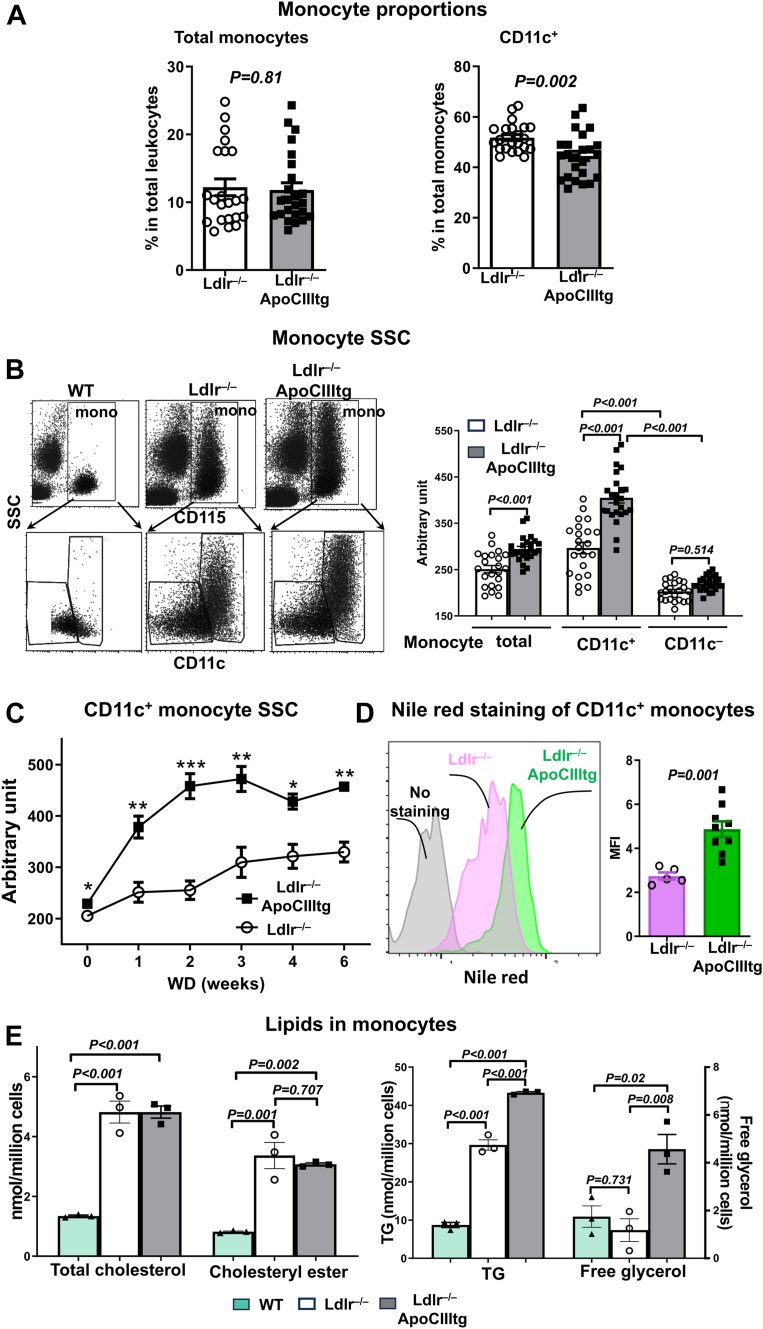
Changes in monocyte and subset proportions and lipid accumulation in *Ldlr*^−/−^apoCIIItg mice. All samples were collected from *Ldlr*^−/−^ApoCIIItg and *Ldlr*^−/−^ mice fed WD for 6 weeks or as indicated otherwise. A: Percentage of total monocytes in total leukocytes and percentage of CD11c^+^ monocytes in total monocytes in blood of mice. B: Representative flow cytometric (FACS) examples of monocytes showing SSC, representing cell granularity and intracellular lipid droplet accumulation, and quantification of monocyte and subset SSC. Data were collected from three independent experiments with 6–8 mice/group in each experiment (A and B). C: Changes in SSC of CD11c^+^ monocytes in mice during WD (n = 4–9 samples/group). D: FACS examples and quantification of Nile Red staining for lipids in CD11c^+^ monocytes in mice (n = 5–9 mice/group). E: Quantification of total cholesterol, cholesteryl ester, TG, and free glycerol in monocytes (n = 3 mice/group). Results were normalized to the total cell number. Data are shown as mean ± SEM. ∗*P* < 0.05, ∗∗*P* < 0.01, ∗∗∗*P* < 0.001 compared to *Ldlr*^−/−^ group.

To determine the role of monocytes in increased atherosclerosis in *Ldlr*^−/−^ApoCIIItg mice with HTG, we examined monocytes and phenotypes. Based on CD11c, we classified mouse monocytes into CD11c^+^, which were mainly Ly-6C^low/intermediate^ (supplemental Fig. S3A), CD36^+^ (supplemental Fig. S3B), TREML4^+^, and CD43^high^ (supplemental Fig. S3C), and CD11c^–^ monocytes, which were largely Ly-6C^high^, CD36^–^, TREML4^–/low^, and CD43^+/low^ (supplemental Fig. S3) (6, 8, 13, 25). Small proportions of both CD11c^+^ and CD11c^–^ monocytes expressed MHCII, but neither subset expressed XCR1 (supplemental Fig. S3C). At 6 weeks on WD, *Ldlr*^−/−^ApoCIIItg mice compared to *Ldlr*^−/−^ controls showed no significant differences in the percentage of total monocytes in total leukocytes but a slight reduction in the percentage of CD11c^+^ monocytes in total monocytes (Fig. 2A).

We then examined lipid accumulation within monocytes, that is, foamy monocyte formation. As previously reported (6, 8, 13, 25), we defined and quantified foamy monocytes by elevated side scatter (SSC, referring to SSC area unless stated otherwise) in FACS analysis (Fig. 2B), which represented increased cell granularity and correlated with inclusion of lipid droplets. Along with increased atherosclerosis, *Ldlr*^−/−^ApoCIIItg mice compared to *Ldlr*^−/−^ controls fed WD for 6 weeks had significant elevations in SSC, indicating increased lipid accumulation, in monocytes (Fig. 2B). In both *Ldlr*^−/−^ApoCIIItg and control mice, CD11c^+^ monocytes were the main foamy monocytes with increased SSC, whereas CD11c^–^ monocytes had much lower SSC, indicating less lipid accumulation (Fig. 2B), consistent with our previous reports in apoE^−/−^ and *Ldlr*^−/−^ mice (6, 8, 13). Compared to *Ldlr*^−/−^ controls, *Ldlr*^−/−^ApoCIIItg mice fed WD had significant increases in SSC values of CD11c^+^ monocytes but not CD11c^–^ monocytes (Fig. 2B). Further analysis showed that SSC values of CD11c^+^ monocytes were slightly but significantly higher in *Ldlr*^−/−^ApoCIIItg mice than control mice fed ND and had greater increases early and persistently in *Ldlr*^−/−^ApoCIIItg mice than controls fed WD (Fig. 2C). Nile Red staining confirmed increased lipid accumulation in CD11c^+^ monocytes in *Ldlr*^−/−^ApoCIIItg mice (Fig. 2D). Lipid composition analysis revealed that compared to WT mice fed ND, both *Ldlr*^−/−^ and *Ldlr*^−/−^ApoCIIItg mice fed WD had significant increases in monocyte total cholesterol and cholesteryl ester levels, with no significant differences between *Ldlr*^−/−^ and *Ldlr*^−/−^ApoCIIItg mice (Fig. 2E). In contrast, while both *Ldlr*^−/−^ApoCIIItg and *Ldlr*^−/−^ mice compared to WT had increased levels of monocyte TG, *Ldlr*^−/−^ApoCIIItg, but not *Ldlr*^−/−^, mice had increased monocyte-free glycerol levels, and *Ldlr*^−/−^ApoCIIItg mice compared to *Ldlr*^−/−^ controls had higher levels of TG and free glycerol in monocytes (Fig. 2E). In summary, compared to hypercholesterolemia in *Ldlr*^−/−^ mice, HTG and combined hyperlipidemia in *Ldlr*^−/−^ApoCIIItg mice induced early and persistent increases in lipid accumulation within monocytes, CD11c^+^ monocytes in particular. Compared to *Ldlr*^−/−^ controls, *Ldlr*^−/−^ApoCIIItg mice had increased levels of TG and free glycerol in monocytes.

### Changes in monocyte phenotypes in *Ldlr*^−/−^ApoCIIItg mice

**Figure 3:**
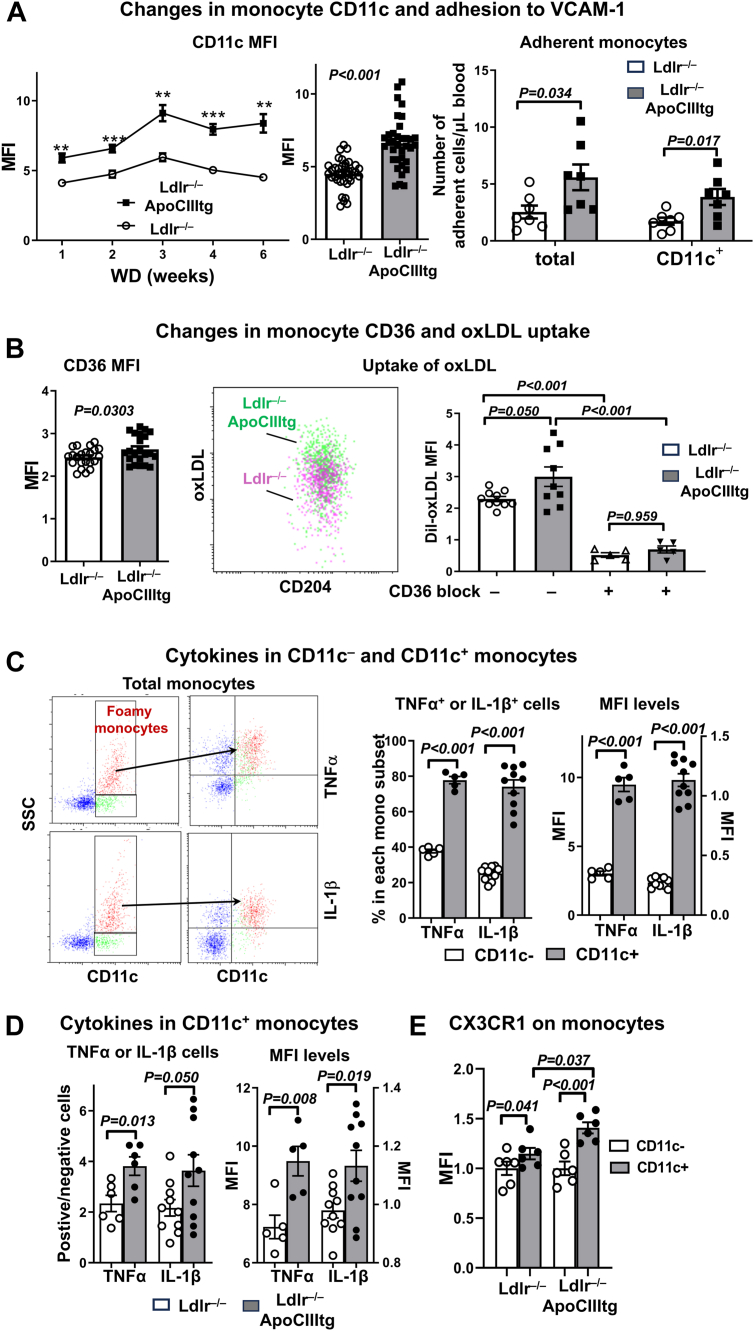
Changes in monocyte and subset phenotypes in *Ldlr*^−/−^apoCIIItg mice. *Ldlr*^−/−^ApoCIIItg and *Ldlr*^−/−^ mice were fed WD for 6 weeks or as indicated otherwise. A: Change in CD11c MFI of foamy (CD11c^+^) monocytes (n = 8–10 mice/group), CD11c MFI of foamy (CD11c^+^) monocytes, and monocyte adhesion to VCAM-1 examined by ex vivo flow adhesion assay. B: Monocyte CD36 MFI and OxLDL uptake ex vivo. Data show representative FACS examples and quantification of OxLDL uptake by CD11c^+^ monocytes. C: Representative FACS examples and quantification of intracellular TNFα and IL-1β in CD11c^+^ and CD11c^–^ monocytes in *Ldlr*^−/−^ApoCIIItg mice. D: Intracellular TNFα and IL-1β in CD11c^+^ monocytes in *Ldlr*^−/−^ApoCIIItg and *Ldlr*^−/−^ mice. E: CX3CR1 MFI on CD11c^+^ and CD11c^–^ monocytes in mice. Data are shown as mean ± SEM. ∗*P* < 0.05, ∗∗*P* < 0.01, ∗∗∗*P* < 0.001 compared to *Ldlr*^−/−^ group (A).

Analysis of monocyte phenotypes showed that with the increased lipid accumulation, foamy monocytes in *Ldlr*^−/−^ApoCIIItg mice compared to those in control mice on WD exhibited early and persistent increases in CD11c levels as indicated by mean fluorescence intensity (MFI, Fig. 3A), which were associated with increased monocyte adhesion to E-selectin/VCAM-1 ex vivo under flow (Fig. 3A). In addition, compared to those in control mice, foamy monocytes in *Ldlr*^−/−^ApoCIIItg mice on WD expressed higher levels of CD36, which were associated with increased monocyte uptake of OxLDL as indicated by higher DiI MFI values after coincubation with DiI-OxLDL ex vivo (Fig. 3B and supplemental Fig. S4). Furthermore, antibody blockade of CD36 significantly decreased DiI-OxLDL uptake by monocytes in both control and *Ldlr*^−/−^ApoCIIItg mice (Fig. 3B).

Analysis of intracellular cytokines showed that in both *Ldlr*^−/−^ApoCIIItg mice (Fig. 3C) and *Ldlr*^−/−^ controls (data not shown), monocytes with elevated SSC (lipid accumulation, CD11c^+^) expressed higher levels of TNFα and IL-1β, indicated by higher proportions of TNFα^high^ and IL-1β^high^ cells and higher MFI levels of TNFα and IL-1β than did monocytes with low SSC (CD11c^–^) (Fig. 3C), indicating that lipid accumulation in monocytes was associated with increased TNFα and IL-1β expression. Importantly, with increased lipid accumulation, CD11c^+^ monocytes in *Ldlr*^−/−^ApoCIIItg mice compared to those in *Ldlr*^−/−^ controls expressed even higher levels of TNFα and IL-1β (Fig. 3D).

Compared to CD11c^–^ (Ly-6C^high^) monocytes, CD11c^+^ (Ly-6C^low^) monocytes expressed higher levels of CX3CR1, which was even higher on CD11c^+^ monocytes in *Ldlr*^−/−^ApoCIIItg mice than in *Ldlr*^−/−^ mice (Fig. 3E).

Taken together, HTG and combined hyperlipidemia in *Ldlr*^−/−^ApoCIIItg mice on WD, with induction of increased monocyte lipid accumulation, enhanced expression levels of CD11c, CD36, TNFα, IL-1β, and CX3CR1 in monocytes, particularly CD11c^+^ monocytes, which exhibited increased adhesion to VCAM-1 and augmented uptake of modified LDL.

### Effects of TGRL on monocyte phenotypes

**Figure 4:**
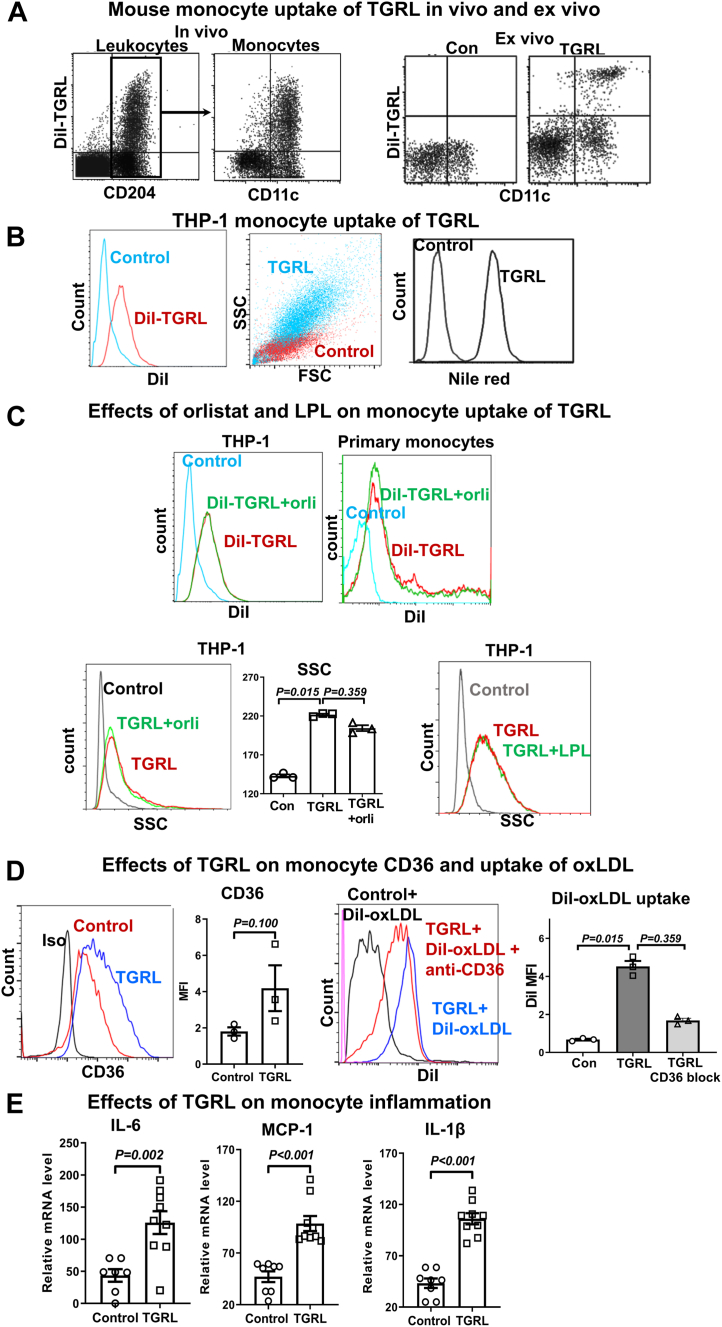
Monocyte uptake of TGRL and phenotypic changes. A: Representative FACS examples of mouse monocyte uptake of TGRL in vivo and ex vivo. Left panel (in vivo): DiI-mTGRL isolated from *Ldlr*^−/−^ApoCIIItg mice and labeled with DiI were intravenously injected into WT mice (recipients), and monocyte uptake of DiI-mTGRL was examined in recipient blood 24 h later after staining for CD204 and CD11c. Right panel (ex vivo): Blood from WT mice was incubated with or without DiI-mTGRL for 3 h, and monocyte uptake of DiI-mTGRL was examined after staining for CD204 and CD11c. Data shown were gated monocytes (CD204^+^). B: Representative FACS examples of THP-1 monocyte uptake of TGRL (indicated by DiI signal after incubation with DiI-TGRL for 4 h) and lipid accumulation (indicated by elevated SSC and Nile Red staining after incubation with TGRL for 48 h). C: Monocyte uptake of TGRL and lipid accumulation with lipoprotein lipase (LPL) inhibition or addition. THP-1 monocytes or blood from healthy humans were incubated with DiI-labeled or unlabeled postprandial TGRL in the presence or the absence of orlistat (orli) for 4 h (upper panels for DiI-TGRL uptake) or 24 h (lower left and middle panels for lipid accumulation). Lower right panel: representative FACS examples of lipid accumulation in THP-1 monocytes treated with TGRL for 48 h in the presence or the absence of exogenous LPL. D: Representative FACS examples and quantification of effects of TGRL treatment on THP-1 CD36 expression and uptake of OxLDL. THP-1 monocytes were treated with TGRL for 48 h and then, after being washed with PBS to remove TGRL, incubated with DiI-OxLDL in the presence or the absence of a CD36 mAb for an additional 4 h. THP-1 expression of CD36 was examined after incubation with TGRL and before incubation with DiI-OxLDL, and THP-1 uptake of DiI-OxLDL was examined after incubation with DiI-OxLDL. E: Effects of TGRL on THP-1 expression of IL-6, MCP-1, and IL-1β examined by quantitative RT-PCR. All representative examples were from ≥3 independent experiments with similar results. Data are shown as mean ± SEM.

Next, we examined direct effects of TGRL on monocyte lipid accumulation and phenotypes. A bolus injection of DiI-labeled TGRL isolated from *Ldlr*^−/−^ApoCIIItg mice into WT mice resulted in TGRL uptake, indicated by DiI signals, specifically in monocytes (CD204^+^) in the circulation. Staining for CD11c showed that CD11c^+^, but few CD11c^–^, monocytes took up TGRL and became DiI^+^ (Fig. 4A). Consistently, incubation with DiI-TGRL (from *Ldlr*^−/−^ApoCIIItg mice) confirmed that monocytes, particularly CD11c^+^ monocytes (from WT mice), took up TGRL ex vivo (Fig. 4A and supplemental Fig. S4).

We then treated THP-1 monocytes with postprandial TGRL isolated from human subjects with metabolic syndrome. Incubation with DiI-TGRL for 4 h resulted in TGRL uptake by THP-1 monocytes indicated by being DiI^+^ (Fig. 4B). Incubation with TGRL for 48 h markedly increased SSC of THP-1 monocytes, indicating lipid accumulation, which was confirmed by Nile Red staining (Fig. 4B). TG hydrolysis by LPL has been implicated in HTG-induced lipid accumulation in tissue macrophages (17). However, adding orlistat to inhibit LPL in the culture system did not largely impact THP-1 or primary monocyte uptake of TGRL or lipid accumulation (Fig. 4C), indicating that LPL-mediated TG hydrolysis may not play a major role in TGRL-/HTG-induced monocyte lipid accumulation. On the other hand, adding LPL did not increase lipid accumulation in THP-1 monocytes treated with TGRL (Fig. 4C).

Treatment with postprandial TGRL tended to increase CD36 levels on THP-1 monocytes and enhanced DiI-OxLDL uptake (Fig. 4D). Antibody blockade of CD36 tended to reduce THP-1 uptake of OxLDL (Fig. 4D), consistently supporting a role of CD36 in monocyte uptake of OxLDL. Furthermore, treatment with postprandial TGRL increased THP-1 expression of cytokines including IL-1β, MCP-1, and IL-6 (Fig. 4E).

Taken together, monocytes took up TGRL in vivo and in vitro/ex vivo, a process mostly independent of LPL, leading to monocyte lipid accumulation and phenotypic changes with increased expression of inflammatory molecules and CD36, which increased monocyte uptake of OxLDL.

### Infiltration of foamy monocytes into atherosclerotic lesions in *Ldlr*^−/−^ApoCIIItg mice

**Figure 5:**
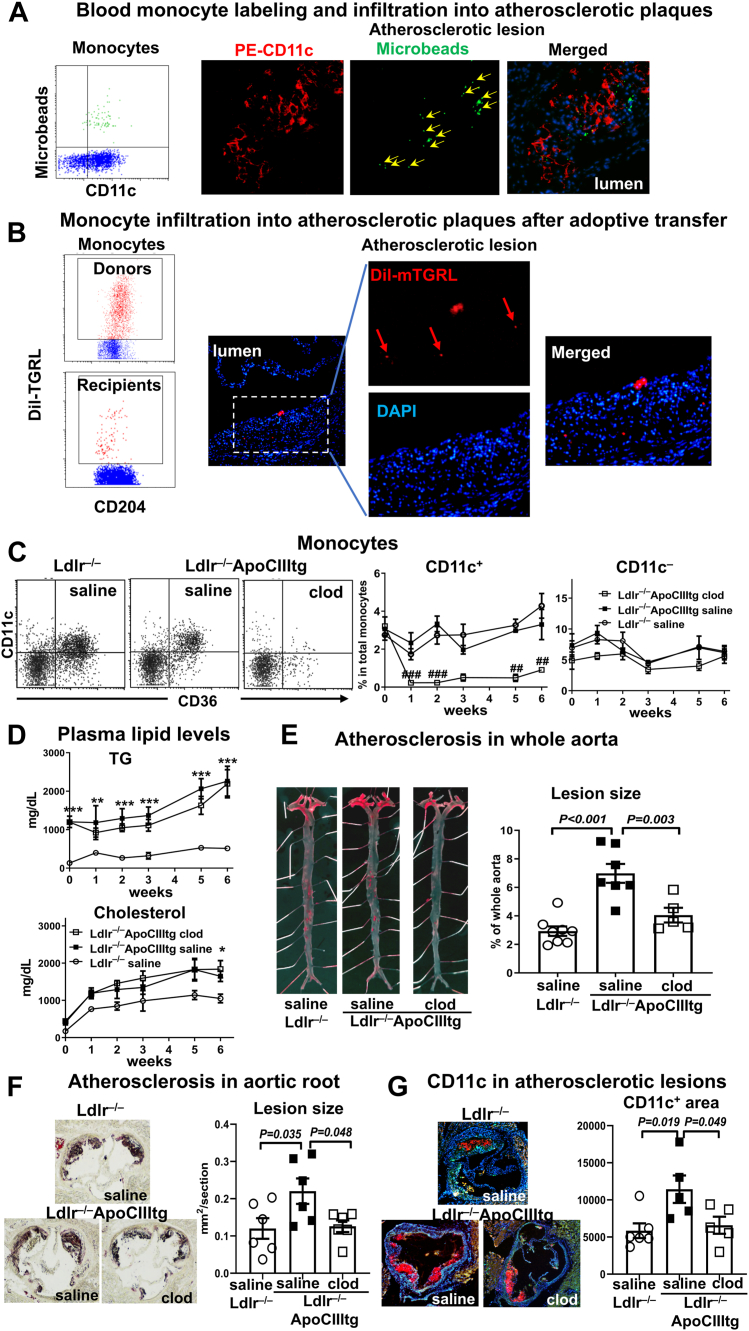
Role of foamy monocytes in atherosclerosis development in *Ldlr*^−/−^apoCIIItg mice. A: Representative FACS example showing labeling of circulating CD11c^+^ (foamy) monocytes with microbeads and histology of aortic sinus showing infiltration of microbead (green)-labeled CD11c^+^ monocytes. *Ldlr*^−/−^ApoCIIItg mice fed WD for 6 weeks received intravenous injection of fluorescent microbeads; labeling of circulating monocytes was examined at 24 h, and infiltration of labeled monocytes was examined at 48 h after microbead injection. B: Representative FACS examples showing DiI-TGRL-labeled monocytes in donor (left) and recipient (right) blood and histology of recipient aortic sinus showing infiltration of DiI-TGRL (red)-labeled monocytes (CD11c^+^, Fig. 4A). DiI-mTGRL was intravenously injected into WT mice (donors). After 24 h, blood was analyzed by FACS, and mononuclear cells were isolated from donors and injected intravenously into recipient *Ldlr*^−/−^ApoCIIItg mice fed WD (12 weeks). Twenty-four hours later, the same recipients received another injection of labeled mononuclear cells from WT and were examined for labeled monocytes in the circulation at 24 h and infiltration of labeled monocytes at 48 h after the second injection of mononuclear cells. C–G: Effects of depletion of CD11c^+^ (foamy) monocytes on atherogenesis in HTG. *Ldlr*^−/−^ApoCIIItg mice fed WD received intravenous injection of low-dose clodrosome (clod) or saline (and *Ldlr*^−/−^ mice received saline) daily for 6 weeks (n = 5–7 mice/group). C: Representative FACS examples showing specific depletion of CD11c^+^ monocytes with clodrosome injection (1 week) and quantification of CD11c^+^ and CD11c^–^ monocytes. D: Plasma total TG and cholesterol levels. E: Representative photographs and quantification of en face Oil Red O staining of whole aorta. F: Representative photographs and quantification of Oil Red O staining in aortic sinus. G: Representative photographs and quantification of CD11c staining in aortic sinus lesions. Data are shown as mean ± SEM. ∗*P* < 0.05, ∗∗*P* < 0.01, ∗∗∗*P* < 0.001 compared to *Ldlr*^−/−^ mice with saline (D); ##*P* < 0.01, ###*P* < 0.001 compared to *Ldlr*^−/−^ApoCIIItg mice with saline (C).

Given the critical role of monocyte infiltration in atherosclerosis development and progression, we assessed monocyte infiltration into atherosclerosis in *Ldlr*^−/−^ApoCIIItg mice in two protocols. In protocol 1, by injection of fluorescent microbeads, CD11c^+^ (Ly-6C^low^) foamy monocytes were specifically labeled in *Ldlr*^−/−^ApoCIIItg mice (Fig. 5A) (8, 9). Examination of atherosclerotic lesions indicated that microbead-labeled foamy monocytes infiltrated into lesions in *Ldlr*^−/−^ApoCIIItg mice (Fig. 5A). In protocol 2, after injection into mice (donors), DiI-TGRL were specifically taken up by monocytes, particularly CD11c^+^ monocytes, leading to CD11c^+^ monocyte labeling with DiI-TGRL (Figs. 5B and 4A). Adoptive transfer of DiI-TGRL-labeled CD11c^+^ monocytes into recipient *Ldlr*^−/−^ApoCIIItg mice showed that at 24 h after a second transfer, DiI-TGRL-labeled CD11c^+^ monocytes were still detectable in the circulation of recipients (Fig. 5B). At 48 h after a second transfer of DiI-TGRL-labeled CD11c^+^ monocytes, infiltration of CD11c^+^ (DiI^+^) monocytes into atherosclerotic plaques was detected in recipients (Fig. 5B). Therefore, both protocols confirmed that CD11c^+^ foamy monocytes infiltrated into atherosclerotic plaques in *Ldlr*^−/−^ApoCIIItg mice.

### Role of CD11c^+^ monocytes in atherosclerosis development in *Ldlr*^−/−^ApoCIIItg mice

To determine the role of foamy monocytes in atherogenesis associated with HTG in combined hyperlipidemia, we specifically depleted CD11c^+^ (foamy) monocytes in WD-fed *Ldlr*^−/−^ApoCIIItg mice (Fig. 5C) by repetitively injecting low-dose clodrosome and then analyzed atherosclerosis (8). Low-dose clodrosome did not significantly impact CD11c^–^ monocytes (Fig. 5C), lesional macrophages (8, 12), or plasma lipid levels (Fig. 5D). *Ldlr*^−/−^ mice with saline injection were also included as a control group. Consistently, plaque size and CD11c^+^ foam cell areas in whole aortas and aortic roots were greater in *Ldlr*^−/−^ApoCIIItg than *Ldlr*^−/−^ mice with saline injection (Fig. 5E–G). Notably, specific depletion of CD11c^+^ monocytes for 6 weeks significantly reduced plaque size and CD11c^+^ foam cell areas in whole aortas and aortic roots in *Ldlr*^−/−^ApoCIIItg mice (Fig. 5E–G), suggesting that CD11c^+^ foamy monocytes played a role in atherogenesis associated with HTG in combined hyperlipidemia.

### Effects of apoCIII ASO treatment on monocyte phenotypes and atherosclerosis in *Ldlr*^−/−^ApoCIIItg mice

**Figure 6:**
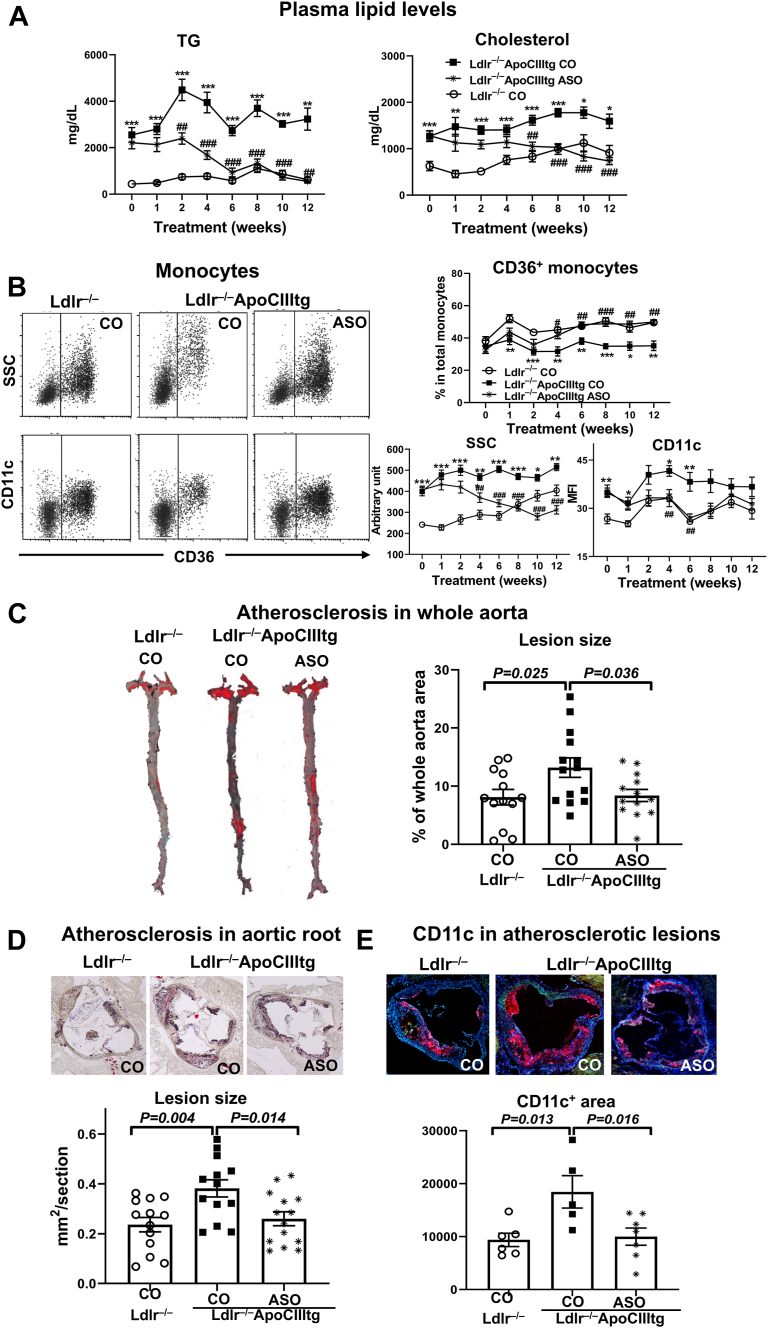
Effects of apoCIII ASO treatment on plasma lipids, monocyte phenotypes, and atherosclerosis in *Ldlr*^−/−^apoCIIItg mice. *Ldlr*^−/−^ApoCIIItg mice fed WD were treated with a GalNac-conjugated ASO against human apoCIII or a GalNac-conjugated CO (and *Ldlr*^−/−^ mice were treated with CO) at 10 mg/kg body weight weekly for 12 weeks. A: Plasma total TG and cholesterol levels (n = 14–19 mice/group from three independent experiments with 4–7 mice/group in each experiment). B: Representative FACS examples showing SSC and CD11c of CD36^+^ and CD36^–^ monocytes (treatment for 4 weeks) and quantification of changes in proportions, SSC, and CD11c MFI of CD36^+^ monocytes with treatment (n = 11–14 mice/group). C: Representative photographs and quantification of en face Oil Red O staining of whole aorta. D: Representative photographs and quantification of Oil Red O staining in aortic sinus. E: Representative photographs and quantification of CD11c staining in aortic sinus lesions. Data are shown as mean ± SEM. ∗*P* < 0.05, ∗∗*P* < 0.01, ∗∗∗*P* < 0.001 compared to *Ldlr*^−/−^ mice with CO (A and B); ##*P* < 0.01, ###*P* < 0.001 compared to *Ldlr*^−/−^ApoCIIItg mice with CO (A and B).

Given the changes in monocytes and atherosclerosis associated with HTG in combined hyperlipidemia, we next tested whether reducing HTG impacted monocytes and atherosclerosis in *Ldlr*^−/−^ApoCIIItg mice. Antisense inhibition of apoCIII with ASO in individuals with HTG significantly reduced HTG (24, 30). Therefore, we determined the effects of this new treatment in *Ldlr*^−/−^ApoCIIItg mice. Treatment with apoCIII ASO versus CO in *Ldlr*^−/−^ApoCIIItg mice did not largely change body weight, liver, epididymal adipose tissue weight (supplemental Fig. S5A), and blood cell counts (supplemental Fig. S5B). As expected, apoCIII ASO treatment in *Ldlr*^−/−^ApoCIIItg mice markedly reduced human apoCIII levels in the liver (supplemental Fig. S6A), induced early and persistent reductions in plasma TG levels, and lowered plasma cholesterol levels (Fig. 6A), with no significant impacts on plasma alanine aminotransferase levels (supplemental Fig. S6B), indicating no significant damage to the liver. Compared to CO, apoCIII ASO tended to reduce insulin sensitivity in *Ldlr*^−/−^ApoCIIItg mice (supplemental Fig. S7).

Consistently, the percentage of CD36^+^ (mostly CD11c^+^ and foamy) monocytes in total monocytes was slightly lower in *Ldlr*^−/−^ApoCIIItg mice with CO injection than in *Ldlr*^−/−^ mice (Fig. 6B). Of note, apoCIII ASO treatment in *Ldlr*^−/−^ApoCIIItg mice largely restored the proportion of CD36^+^ monocytes to a level similar to that in *Ldlr*^−/−^ mice (Fig. 6B). Strikingly, treatment with apoCIII ASO versus CO in *Ldlr*^−/−^ApoCIIItg mice decreased monocyte lipid accumulation, indicated by reduced SSC, early and persistently and reduced CD11c MFI of CD36^+^ monocytes to levels similar to those in *Ldlr*^−/−^ mice (Fig. 6B). Analysis of monocyte lipids showed that apoCIII ASO compared to CO treatment in *Ldlr*^−/−^ApoCIIItg mice decreased monocyte total cholesterol and cholesteryl ester levels (supplemental Fig. S8). Importantly, compared to CO, apoCIII ASO treatment in *Ldlr*^−/−^ApoCIIItg mice fed WD significantly reduced atherosclerosis as indicated by smaller plaque size in whole aortas and aortic roots and smaller CD11c^+^ foam cell areas in aortic root plaques (Fig. 6C–E). These data suggest that apoCIII ASO treatment lowered plasma TG and cholesterol levels, improved circulating monocyte phenotypes, and reduced atherosclerosis in *Ldlr*^−/−^ApoCIIItg mice with HTG and combined hyperlipidemia.

Notably, treatment of *Ldlr*^−/−^ mice with anti-mouse apoCIII ASO compared to CO significantly reduced plasma apoCIII levels and tended to reduce plasma TG levels, with no significant impacts on plasma cholesterol levels (supplemental Fig. S9A). Analyses of monocytes showed that compared to CO, apoCIII ASO treatment of *Ldlr*^−/−^ mice did not significantly change monocyte lipid accumulation indicated by SSC and TG and cholesterol content (supplemental Fig. S9B).

## Discussion

We report our novel observations that elevated TGRL in HTG enhance foamy monocyte formation in the circulation, with increased monocyte accumulation of lipids, particularly TG, and induce monocyte phenotypic changes in mice with hypercholesterolemia and that foamy monocytes contribute to acceleration of atherogenesis in mice with HTG and combined hyperlipidemia. Importantly, we also report that treatment with apoCIII ASO reduces monocyte lipid accumulation, improves monocyte phenotypes, and decelerates atherosclerosis progression in mice with combined hyperlipidemia.

HTG, particularly in combined hyperlipidemia (with elevated LDL-cholesterol or non-HDL-cholesterol), increases risk for ASCVD. Consistently, HTG accelerates atherogenesis in mice with hypercholesterolemia (28, 29). However, how HTG in combined hyperlipidemia contributes to atherosclerosis remains poorly understood. Our study revealed interaction of TGRL with circulating monocytes, leading to foamy monocyte formation and monocyte phenotypic changes, as a potential novel mechanistic link between HTG and atherosclerosis in combined hyperlipidemia. Previous studies from our laboratory and others’ revealed formation of lipid-laden foamy monocytes in the circulation of mice and humans with hypercholesterolemia and their contributions to atherosclerosis in hypercholesterolemia (6, 8, 13, 14). HTG in humans with metabolic syndrome or induced by a single high-fat meal also increases monocyte lipid accumulation (15, 16, 18, 19, 20). In the current study, we observed that HTG in mice with hypercholesterolemia significantly enhanced lipid accumulation in circulating monocytes, particularly CD11c^+^/Ly-6C^low/intermediate^ monocytes, which are analogous to human CD16^+^ intermediate and nonclassical monocytes that correlate positively with ASCVD (31, 32, 33). Mechanistically, monocytes take up TGRL in vivo and in vitro and become foamy, a process mostly independent of LPL, consistent with a previous report (17) and different from that in tissue macrophages, which largely rely on LPL for TGRL-induced lipid accumulation (17).

Increased lipid accumulation in foamy monocytes in *Ldlr*^−/−^ApoCIIItg mice with HTG in combined hyperlipidemia was associated with intensified monocyte phenotypic changes. CD11c, a β2 integrin highly expressed on foamy monocytes, mediates foamy monocyte adhesion and contributes to atherosclerosis development in hypercholesterolemia (6, 19). HTG in *Ldlr*^−/−^ApoCIIItg mice further upregulated CD11c levels on foamy monocytes, with enhanced monocyte adhesion to VCAM-1, a process critical for monocyte transendothelial migration and atherosclerosis development. Increased lipid accumulation in monocytes of *Ldlr*^−/−^ApoCIIItg mice was also associated with increased monocyte inflammation, with elevated expression of TNFα and IL-1β. This observation, as well as our in vitro studies and a previous report (34), was consistent with the clinical evidence that TGRL compared to LDL correlate better with inflammation (35, 36, 37), a process that plays a pivotal role in ASCVD (35). In addition, foamy monocytes in *Ldlr*^−/−^ApoCIIItg mice expressed elevated levels of CD36, which enhanced monocyte uptake of OxLDL. Therefore, one of the mechanisms by which HTG in combined hyperlipidemia enhances atherogenesis may also involve acceleration of monocyte/macrophage uptake of modified LDL, a process important for foam cell formation and atherogenesis.

All the discussed changes in foamy monocyte phenotypes may contribute to HTG-associated increases in atherogenesis in *Ldlr*^−/−^ApoCIIItg mice. The slight reduction in the proportion of CD11c^+^ (CD36^+^ and Ly-6C^low^) foamy monocytes in the circulation of *Ldlr*^−/−^ApoCIIItg mice with HTG in combined hyperlipidemia was possibly owing to increased extravasation of this monocyte subset into tissues (38), including atherosclerotic plaques, supporting a role of foamy monocytes in atherogenesis. Indeed, foamy monocytes infiltrated into atherosclerotic plaques and played an important role in atherogenesis with HTG in combined hyperlipidemia as evidenced by reduction of atherosclerosis with specific depletion of CD11c^+^ (foamy) monocytes in *Ldlr*^−/−^ApoCIIItg mice. It is recognized that by transcytosis across the endothelial layer, TGRL remnants can enter arterial walls, where TGRL remnants interact with macrophages and contribute to atherogenesis (39). However, because of their size, large TGRL may have difficulty penetrating the endothelial layer and entering arterial walls. TGRL/remnant-monocyte interactions in the circulation, leading to foamy monocyte formation and infiltration into arterial walls, may serve as an additional pathway whereby HTG accelerates ASCVD.

ApoCIII has been previously shown to induce monocyte inflammation and enhance monocyte adhesion to endothelium (40, 41). It remains to be determined whether increased lipid accumulation or increased apoCIII per se drives the exacerbation of monocyte phenotypic changes in *Ldlr*^−/−^ApoCIIItg mice. While one study attributed the inflammatory role of TGRL to apoCIII residing in the TGRL (40), another study indicated that apoCIII may induce inflammation only in its delipidated form (42), which occurs at low levels and may not be sufficient to drive inflammation in the circulation of mice, including ApoCIIItg mice. Therefore, it is more likely that increased lipid accumulation rather than apoCIII exacerbated phenotypes of circulating monocytes in *Ldlr*^−/−^ApoCIIItg mice. Nonetheless, the exact mechanisms whereby TGRL increase lipid accumulation in monocytes and increased lipid accumulation drives monocyte phenotypic changes remain to be determined.

Clinical trials have shown the efficacy of apoCIII ASO in lowering plasma TG and also, to a lesser extent, lowering total cholesterol, mainly non-HDL-cholesterol, in subjects with HTG (24, 30, 43). Our study showed that apoCIII ASO treatment in *Ldlr*^−/−^ApoCIIItg mice resulted in early and persistent reductions in plasma TG levels and modest reductions in plasma cholesterol levels, which were associated with decreased monocyte lipid accumulation, improved monocyte phenotypes, and reduced atherosclerosis progression. These observations are consistent with recent studies showing that apoCIII ASO treatment reduces atherosclerosis progression or improves atherosclerotic plaque stability in mice with type 1 diabetes and elevated apoCIII by reducing apoCIII (44) or in mice with HTG by reducing TG (45). In contrast, mice with no elevations of apoCIII or without severe HTG did not show responses to apoCIII ASO treatment in atherogenesis (44, 45). Consistently, we report that apoCIII ASO treatment did not alter monocyte lipid accumulation and TG and cholesterol content in *Ldlr*^−/−^ mice. Additional to the clinical evidence for HTG-lowering effects of apoCIII ASO, these animal studies provide experimental support for the potential benefit of this new therapy for ASCVD prevention in individuals with HTG and/or elevated apoCIII.

A limitation of our current study is that we were unable to dissect fully the role of apoCIII from that of TGRL particles in atherogenesis using in vivo mouse models. ApoCIII and TG levels are highly correlated in mice and humans (4, 46, 47, 48), and lowering TG levels by diet changes or medications also lowers apoCIII levels (24, 30, 47). Furthermore, apoCIII is transferable among different lipoprotein particles (49), making it more difficult to study the role of apoCIII independent of TGRL in vivo. Nonetheless, it is well recognized that apoCIII plays a major role in TG/TGRL metabolism through LPL-dependent and -independent mechanisms (47). Most previous studies suggest that apoCIII may contribute to atherogenesis or ASCVD indirectly, via effects on TGRL metabolism (4, 48, 50, 51), consistent with our discussion of monocyte phenotypic changes above. Another limitation is that only male mice were used in our study, and the observations made in this study cannot be directly applied to female mice.

In conclusion, our study supports a hypothesis that elevated levels of TGRL in HTG and combined hyperlipidemia enhance circulating monocyte lipid accumulation and exacerbate monocyte phenotypic changes, with increases in monocyte inflammation, infiltration into arterial walls, and uptake of modified LDL and in foam cell formation, thereby accelerating atherosclerosis. ASO inhibition of apoCIII lowers plasma TG and cholesterol levels, improves monocyte phenotypes, and reduces atherosclerosis associated with HTG in combined hyperlipidemia. Therefore, apoCIII ASO treatment has potential for preventing ASCVD in individuals with combined hyperlipidemia and high ASCVD risk.

## Data availability

All data are contained within the article and supplemental data.

## Supplemental data

This article contains supplemental data (8, 13, 15, 23, 24, 25, 26, 27).

## Conflict of interest

Dr Ballantyne has received grant/research support through the institution from Ionis Pharmaceuticals and Arrowhead and consultant fees from Ionis Pharmaceuticals and Arrowhead. Dr Mullick and Dr Crooke are employees of Ionis Pharmaceuticals and provided the GalNac-conjugated ASOs against human or mouse apoCIII and CO used in this study. Ionis Pharmaceuticals did not provide financial support for this study. All other authors declare that they have no conflicts of interest with the contents of this article.
