# Supplementary material for: Foamy monocytes and atherogenesis in mice with combined hyperlipidemia and effects of antisense knockdown of apoCIII
Source: J Lipid Res. 2025 Feb 21;66(4):100763. doi: 10.1016/j.jlr.2025.100763 (PMC11981816; doi:10.1016/j.jlr.2025.100763)
Supplement: Supplemental data [file mmc1.pdf]

Supplemental Materials

Foamy monocytes and atherogenesis in mice with combined hyperlipidemia and effects of antisense knockdown of apoCIII

Xueying Peng[a,b], PhD, Zeqin Lian[b], PhD, Veronica O'Brien[b], BS, Jing Xiao[b], PhD, Benjamin A. Litchfield[b], BS, Xiao-Yuan Dai Perrard[b], MD, Lu Xu[b], MD, PhD, Jing Ni[b], MD, PhD, Aparna Mukherjee[b], MS, Timothy Simmons[b], BS, Henry Dong[c], PhD, Adam E. Mullick[d], PhD, Rosanne Crooke[d], PhD, Henry J. Pownall[e], PhD, Scott I. Simon[f], PhD, Christie M. Ballantyne[b,g], MD, Huaizhu Wu[b,*], MD

[a]Department of Clinical Pharmacy, Key Laboratory of Clinical Cancer Pharmacology and Toxicology Research of Zhejiang Province, Affiliated Hangzhou First People's Hospital, Westlake University School of Medicine, Hangzhou, Zhejiang, P.R. China;
[b]Department of Medicine and [g]Center for Cardiometabolic Disease Prevention, Baylor College of Medicine, Houston, TX, USA;
[c]Department of Pediatrics, Children's Hospital of Pittsburgh UPMC, University of Pittsburgh School of Medicine, Pittsburgh, PA, USA;
[d]Ionis Pharmaceuticals, Carlsbad, CA, USA;
[e]Center for Bioenergetics, Houston Methodist Research Institute, Houston, TX, USA;
[f]Department of Biomedical Engineering, University of California, Davis, CA, USA.

***Correspondence:** Huaizhu Wu, Department of Medicine, Baylor College of Medicine, One Baylor Plaza, MS BCM285, Houston, TX, USA 77030. Phone: 714-798-4141. Email: hwu@bcm.edu

Supplemental Materials and Methods

Animals and diet

All lines of mice were developed on C57BL/6J background and maintained in a light-, temperature-, and humidity-controlled environment at the Baylor College of Medicine animal facility. Male $Ldlr^{-/-}$ApoCIIItg ($Ldlr^{-/-}ApoCIII^{+}$) mice and $Ldlr^{-/-}$ controls ($Ldlr^{-/-}ApoCIII^{-}$ littermates) generated by crossing $Ldlr^{-/-}$ (strain #002207, The Jackson Laboratory, Bar Harbor, ME) with ApoCIIItg mice (strain #006907, The Jackson Laboratory) were used for animal studies, which were approved by the Institutional Animal Care and Use Committee of Baylor College of Medicine. After being weaned, mice were fed a normal laboratory diet (ND; 5% fat [w/w]; Rodent Diet 5010, LabDiet, St. Louis, MO) and, at age 8 weeks, switched to western high-fat diet (WD) (21% milkfat [w/w], 0.2% cholesterol; Dyet 112734, Dyets, Inc., Bethlehem, PA) and maintained on WD for 6–13 weeks, during which mice were weighed weekly. Blood was collected by facial vein puncture at times indicated in Results or by cardiac puncture when mice were euthanized via inhalation of 5% or greater isoflurane (USP, NDC: 11695-6777-2, Covetrus, Portland, ME), which was continued until 1 minute after breathing stopped, followed by cervical dislocation. To measure plasma total cholesterol and triglyceride (TG) levels, plasma was isolated from EDTA-anticoagulated blood by centrifugation at 4°C at 1500g for 10 minutes and cholesterol and TG concentrations were determined by standard enzymatic assays (Wako Diagnostics, Richmond, VA) following manufacturer’s instructions. After mice were euthanized, aorta and heart were collected and fixed in 4% paraformaldehyde in phosphate-buffered saline (PBS) or embedded in optimal cutting temperature compound (Sakura Finetek, Torrance, CA). Other tissues including liver and epididymal adipose tissue were also collected and weighed. In addition, insulin tolerance test was performed in a cohort of mice fed WD (for 6 weeks) after fasting for 6 hours as described

previously (1).

Human subjects and TG-rich lipoprotein isolation

Human subjects with metabolic syndrome were recruited, and blood was collected using BD Vacutainer K2EDTA (366643; Becton, Dickinson and Company, Franklin Lakes, NJ) at 5 hours after intake of a high-fat meal (containing ~900 kcal, ~51% of which was from fat and ~23% from saturated fat) (2). The study was performed in compliance with the principles of the Declaration of Helsinki under the protocol #H-21418, which was approved by the Institutional Review Board of Baylor College of Medicine, and all participants provided written informed consent prior to participation. Blood was spun at room temperature for 10 minutes at 1500g. After centrifugation, plasma was transferred to ultracentrifuge tubes (Beckman-Coulter) and centrifuged at 280,000g for 18 hours at 4°C. TG-rich lipoproteins (TGRL) were harvested by aspirating the top layer of plasma ($\rho<1.0063$ g/mL), with precaution to avoid endotoxin contamination. In some cases, TGRL were labeled with DiI (SigmaAldrich, St. Louis, MO) by incubation with DiI solution (3.7 mg/mL in dimethyl sulfoxide [SigmaAldrich]) at room temperature for 30 minutes followed by overnight dialysis in sterile PBS (3, 4). DiI-TGRL were stored at 4°C, protected from light, and used within 2 weeks.

Treatment of mice with antisense oligonucleotide against apoCIII

A GalNac-conjugated antisense oligonucleotide (ASO) against human apoCIII and a GalNac-conjugated control oligonucleotide (CO) were provided by Ionis Pharmaceuticals (Carlsbad, CA) (5). Male Ldlr$^{-/-}$ApoCIIItg mice were fed WD for 1 week; then mice with corresponding TG levels were randomized into CO and ASO treatment groups, which received weekly subcutaneous

injection of 10 mg/kg CO and ASO, respectively. An Ldlr$^{-/-}$ control group receiving weekly subcutaneous injection of CO was also included. During CO and ASO treatment, blood was collected weekly or biweekly (see Results) via facial vein puncture into EDTA-coated tubes for analyses of plasma TG and cholesterol and monocyte phenotypes. After treatment for 12 weeks, mice were euthanized, and aorta and heart were collected for analyses of atherosclerosis. Complete blood count was also performed at the end of treatment. A separate cohort of Ldlr$^{-/-}$ApoCIIItg mice fed WD were treated with CO or ASO for 6 weeks, and monocytes were isolated and analyzed for TG and cholesterol content. In another cohort, Ldlr$^{-/-}$ mice fed WD were treated with GalNac-conjugated ASO against mouse apoCIII or CO via weekly subcutaneous injection at 15 mg/kg for 6 weeks, during which plasma levels of TG and cholesterol were monitored. At the end of the treatment period, monocyte lipids were quantified using commercial assay kits (see below) and plasma apoCIII levels were also measured using a mouse apoCIII ELISA kit (Avantor, Inc., USA) following the manufacturer's instructions.

Plasma lipoprotein profiles

Plasma was collected from EDTA-anticoagulated mouse blood after centrifugation at 1500g for 10 minutes at 4°C. Plasma lipoproteins were isolated by flotation at d=1.21 g/mL and separated into subclasses by size exclusion chromatography over Superose HR6 (GE HealthCare, Chicago, IL). Briefly, 200 to 500 µL of samples was injected, and the effluent was monitored by absorbance at 280 nm and collected as 1-mL fractions (3, 6). Cholesterol and TG concentrations in each fraction were determined enzymatically using commercial kits (Wako Diagnostics) following manufacturer's instructions.

Antibodies and fluorescence-activated cell sorter (FACS) analysis of circulating monocytes

Monoclonal antibodies (mAbs) to mouse antigens, including CD115 (PE, AFS98, ThermoFisher Scientific, Waltham, MA; or BV421, AFS98, Biolegend, San Diego, CA), CD11c (PerCP-Cy5.5, N418, ThermoFisher Scientific; or APC or FITC, N418, Biolegend), CD36 (FITC, MF3, Bio-Rad Laboratories, Hercules, CA), CD204 (FITC, 2F8, Bio-Rad Laboratories), Ly-6C (APC, HK1.4, ThermoFisher Scientific), CX3CR1 (FITC, SA011F11, Biolegend), MHCII (I-A/I-E; PE, M5/114, BD Biosciences, San Jose, CA), CD43 (PE, S11, Biolegend), TREML4 (PE, 16E5, Biolegend), XCR1 (PE, ZET, Biolegend), tumor necrosis factor α (TNFα; PE, MP6-XT22, ThermoFisher Scientific), and interleukin-1β (IL-1β; PE, NJTEN3, ThermoFisher Scientific), with appropriate isotype-negative controls, were used for FACS analysis of monocytes. For cell surface staining, 50 mL EDTA-anticoagulated mouse blood was diluted with 50 mL PBS supplemented with 0.5% bovine serum albumin (SigmaAldrich) and then incubated with various combinations of fluorescence-conjugated mAbs or with appropriate isotype-negative controls on ice for 20 minutes, followed by lysis of red blood cells and fixation using BD Lysing solution (BD Biosciences) and then washed twice with PBS. For nile red staining, after cell surface staining with mAbs, red blood cells were lysed with RBC lysis buffer (Biolegend) and the samples were then incubated with nile red (0.1 µM, ThermoFisher Scientific) on ice for 20 minutes and washed twice with PBS. For intracellular staining of cytokines, 100 µL EDTA blood was mixed with 100 mL RPMI-1640 complete medium (ThermoFisher Scientific) and incubated with 5 µg/mL lipopolysaccharide (SigmaAldrich) plus Golgi plug (BD Biosciences) for 4 hours at 37°C. Then the blood samples were incubated with 2 mL RBC lysis buffer at room temperature for 15 minutes to lyse red blood cells. After being washed with PBS, the samples were stained for cell surface markers as described above and then for TNFα or IL-1β following the intracellular staining protocol of BD

Cytofix/Cytoperm Plus kit (BD Biosciences). All stained samples were finally resuspended in 2% paraformaldehyde in PBS. Data were collected on a BD LSRII (BD Biosciences) or Beckman Coulter CytoFLEX (Beckman Coulter, Brea, CA) Flow Cytometer and analyzed using Kaluza (Beckman Coulter) or FlowJo (Tree Star Inc., Ashland, OR) software (3, 6). Total leukocytes were first gated based on forward scatter area (FSC-A) vs side scatter area (SSC-A). Within the leukocyte gate, doublets were excluded based on FSC-A vs forward scatter height (FSC-H). Then leukocyte singlets were selected based on side scatter width (SSC-W) vs side scatter height (SSC-H). Total monocytes were defined as $CD115^+$ and/or $CD204^+$ cells within leukocyte singlets. Monocyte subsets were identified using combination staining for CD115, CD11c, CD36, and Ly-6C, with corresponding isotypes as negative controls (Supplemental Figure S1).

Detection of cellular TG and cholesterol in mouse monocytes

TG, free glycerol, total cholesterol, and cholesteryl esters in mouse monocytes were measured using the Triglyceride-Glo assay kit (Promega, Madison, WI) and Cholesterol/Cholesterol Ester-Glo assay kit (Promega) according to the manufacturer's instructions (7). Briefly, monocytes were isolated from mouse blood by enrichment of white blood cells using RosetteSep™ DM-M (StemCell Technologies, Vancouver, BC) followed by negative selection using PE-conjugated mAbs against mouse CD3 (17A2, Biolegend), CD19 (1D3, Biolegend), and Ly-6G (1A8, Biolegend) and separation using mouse anti-PE-nanobeads (Biolegend). Cells (25,000 in 50 µl lysis solution) were added to each well. Glycerol and cholesterol content was measured in cell lysates and normalized to the cell number.

Mouse monocyte uptake of TGRL and oxidized low-density lipoprotein

Mouse TGRL (mTGRL) were isolated from EDTA plasma of Ldlr$^{-/-}$ApoCIIItg mice fed WD (6–12 weeks) by ultracentrifugation and labeled with DiI as described above (3, 4). To examine mouse monocyte uptake of TGRL or oxidized low-density lipoprotein (oxLDL) ex vivo, blood was collected from mice into heparin-coated tubes via facial vein or cardiac puncture. Heparinized blood was washed with PBS to remove endogenous lipoproteins, and incubated with DiI-mTGRL (at 200 mg/dL TG) or Dil-oxLDL (0.05 mg protein/mL, Kalen Biomedical, Montgomery Village, MD) in RPMI-1640 medium at 37°C for 3 hours, with an anti-mouse CD36 mAb (4 μg/mL) (JC63.1, Abcam, Cambridge, UK) or an isotype-negative control. After red blood cells were lysed with RBC lysis buffer, the samples were stained for CD204 and CD11c as described above. Monocyte uptake of DiI-TGRL or DiI-oxLDL was evaluated by FACS analysis (3, 4). In addition, after staining for CD204 and CD11c, an aliquot of the samples was also fixed with RBC Lysis/Fixation Solution (Biolegend), stained with DAPI for cell nuclei, and visualized by a Zeiss LSM780 confocal microscope (Zeiss Microscopy, Oberkochen, Germany).

To examine monocyte uptake of TGRL in vivo, DiI-mTGRL (equivalent to 100 mg/dL TG) were injected into mice at 0.3 mL/mouse via tail vein. After 24 hours, blood was collected from mice and monocyte uptake of TGRL was analyzed by FACS after staining for CD204 and CD11c (4, 8).

Monocyte adhesion assay

The chip microfluidic assembly and monocyte adhesion assay were performed as previously reported (3). Briefly, clean coverslips that were coated with mouse recombinant vascular cell adhesion molecule–1 (VCAM-1)–Fc chimera and E-selectin–Fc chimera (R&D Systems, Minneapolis, MN) were assembled with a 4-channel polydimethylsiloxane device and kept on a

37°C surface. Heparinized blood (0.1 mL) from mice was incubated with PE–anti-mouse CD115 and FITC–anti-mouse CD11c (N418, Biolegend) at room temperature for 20 minutes and then diluted 1:3 in PBS containing calcium and magnesium. Sixty microliters of diluted blood was introduced into the channel at a flow rate that produced a shear stress of 2 dynes/cm^2 at the fluid–glass interface. Blood was perfused for 5 minutes followed by lysis of red blood cells and fixation of the sample on the coverslip using RBC Lysis/Fixation solution (Biolegend) and mounting with ProLong Gold medium containing DAPI (ThermoFisher Scientific). The number of adherent monocytes was counted and normalized by total infused monocyte number.

In vivo monocyte trafficking

To examine monocyte trafficking in Ldlr$^{-/-}$ApoCIIItg mice, 2 protocols were used. In protocol 1, a bolus (0.2 mL/mouse) of fluorescent microbeads (Fluoresbrite® YG Carboxylate Microspheres, Polysciences, Inc., Brentwood, TN) (1:5 dilution in PBS) was injected intravenously into Ldlr$^{-/-}$ApoCIIItg mice fed WD (6 weeks) to specifically label CD11c$^+$ (Ly-6C^{low}) monocytes (4, 6). In protocol 2, DiI-mTGRL (equivalent to 100 mg/dL TG) were intravenously injected into C57BL/6J wild-type (WT) mice (donors) at 0.3 mL/mouse. After 24 hours, whole blood was collected from donors via cardiac puncture into EDTA-coated tubes. A 50-mL aliquot of blood was used for FACS analysis after staining for CD204 and CD11c to confirm monocyte uptake of Dil-TGRL, which also labeled monocytes (see Results). The rest of the blood was used to isolate mononuclear cells by gradient centrifugation using Histopaque 1083 (SigmaAldrich) following manufacturer's instructions. After being washed, isolated mononuclear cells were injected intravenously into recipient Ldlr$^{-/-}$ApoCIIItg mice fed WD (12 weeks) at 2.3 × 10^6 cells (in ~0.2 mL saline) per recipient. Twenty-four hours later, the same recipients received another injection of labeled WT

mononuclear cells at 2.3 × 10^6 cells per recipient. At 48 hours after microbead injection (protocol 1) or second injection of mononuclear cells (protocol 2), hearts of Ldlr$^{-/-}$ApoCIIItg mice were collected and sectioned. The sections were then stained for CD11c (protocol 1, see the following) and nuclei (using DAPI, protocols 1 and 2), and images were captured using an EVOS fl Fluorescence Microscope (Life Technologies, Carlsbad, CA) to examine microbead- or DiI-TGRL–labeled monocyte trafficking into lesions.

Monocyte depletion

CD11c$^+$ (mainly CD36$^+$ and Ly-6C^{low}) monocytes were depleted in Ldlr$^{-/-}$ApoCIIItg mice using low-dose clodrosome (Encapsula NanoScience LLC., Nashville, TN) (4). Concurrent with WD feeding, Ldlr$^{-/-}$ApoCIIItg mice were randomized into 2 groups, which received daily intravenous injections of diluted clodrosome (0.1 mL/mouse, 1:5 dilution in saline) or saline alone for 6 weeks. An Ldlr$^{-/-}$ control group receiving daily intravenous injections of saline alone was also included. During injections, blood was collected weekly from mice via facial vein puncture into EDTA-coated tubes to examine monocytes and plasma lipid levels. After 6 weeks, mice were euthanized at 24 hours after the final clodrosome injection. Heart and whole aorta were collected for analyses of atherosclerosis.

Atherosclerotic lesion analysis

We adhered to the guidelines for experimental atherosclerosis studies as described in the American Heart Association scientific statement (9). Briefly, mouse aortas were harvested and fixed in 4% paraformaldehyde in PBS. After removal of outside fat and connecting tissue, aortas were cut and pinned longitudinally on a black pad and stained with oil red O (0.5 g/100 mL 60% 2-propanol,

SigmaAldrich). Digital images were captured with a Nikon camera (Nikon Instruments, Melville, NY). Atherosclerotic plaque areas that were oil red O–positive were quantitated using ImageJ analysis software and expressed as percentage relative to the whole aortae (3, 4, 6).

To analyze atherosclerosis in the aortic root, mouse hearts embedded in optimal cutting temperature medium were sectioned using a freezing microtome (Leica CM3050 S, Leica Biosystems, Deer Park, IL) by cutting serial cross sections at 5 μm/section starting from the appearance until the disappearance of the leaflets of the aortic valves. The sections were stained with oil red O to quantify lipid areas in atherosclerotic plaques. For fluorescent immunostaining, the sections were fixed in 4% paraformaldehyde in PBS for 5 minutes at room temperature. After being blocked with 2.5% bovine serum albumin in PBS for 30 minutes, aortic sections were stained with a PE-conjugated anti-CD11c mAb (N418, ThermoFisher Scientific) (or a corresponding isotype-negative control) by coincubation overnight at 4°C. Then, the sections were washed and mounted with ProLong Gold medium containing DAPI (ThermoFisher Scientific). For immunohistochemistry staining, the sections were fixed with cold acetone for 5 minutes and then washed with distilled water. After being blocked with 2.5% normal horse serum (Vector Laboratories, Newark, CA) for 30 minutes, the sections were incubated with a primary anti–human apoCIII antibody (ab7619, goat IgG, or a goat IgG isotype-negative control; Abcam) at 5 mg/mL in 2.5% normal horse serum overnight at 4°C. The sections were washed in PBS for 5 minutes and then processed for immunohistochemistry staining using the ImmPRESS HRP Horse Anti-Goat IgG Detection Polymer Kit and the ImmPACT DAB Substrate Kit (Vector Laboratories) following the manufacturer’s instructions. After this staining, the sections were counterstained with hematoxylin and mounted with VectaMount (Vector Laboratories) (8). Images were captured using a microscope coupled with NIS-Elements software (Nikon Eclipse Ci) or an EVOS fl Fluorescence

Microscope and analyzed using ImageJ software or Adobe Photoshop by at least 2 investigators in a blinded manner (3, 4, 6). Imaging parameters and software setup were constant for all photomicrograph acquisitions in an experiment. For each sample, 6 sections at 200-μm intervals from the aorta root (whole length of aortic valve) were used for quantification of the plaque size. Data were presented as the average of the 6 sections per sample. The lumen side was selected as the region of interest, which was recognized as the space inside elastic fibers of the aorta. The area, of which the color intensity was higher than the threshold justified by the negative-control section, was selected as the positive-staining region.

Cell culture

To examine human monocyte uptake of TGRL, THP-1 monocytes (ATCC, Manassas, VA) or blood from healthy subjects (after being washed with PBS to remove endogenous lipoproteins) were incubated with DiI-human TGRL at 200 mg/dL TG in the presence or absence of 100 μM orlistat (a lipase inhibitor; SigmaAldrich) in RPMI-1640 medium for 4 hours at 37°C and then analyzed by FACS (after staining for CD14 in human blood) as described above. THP-1 monocytes were also treated with unlabeled human TGRL (200 mg/dL TG) in the presence or absence of 100 μM orlistat or, in separate experiments, of 2 units/mL lipoprotein lipase (LPL; SigmaAldrich) in RPMI-1640 medium (supplemented with 3% fatty acid–free low-endotoxin bovine serum albumin [SigmaAldrich]) for 24–48 hours at 37°C. Monocyte lipid accumulation and phenotypic changes were examined by FACS and quantitative reverse transcriptase polymerase chain reaction (RT-PCR).

In separate experiments, THP-1 monocytes were treated with unlabeled TGRL (200 mg/dL TG) for 48 hours and then, after being washed with PBS to remove TGRL, incubated with DiI-

oxLDL (0.05 mg protein/mL) in the presence of a CD36 mAb (4 µg/mL, FA6-152, or an isotype-negative control, StemCell Technologies, Vancouver, BC) for an additional 4 hours at 37°C. After being washed twice with PBS, the samples were analyzed by FACS for monocyte uptake of DiI-oxLDL.

RNA isolation and quantitative RT-PCR

Total RNA was isolated from tissues or cells using TRIzol Reagent (ThermoFisher Scientific) and an RNA Miniprep Kit (Zymo Research, Irvine, CA) following manufacturers' instructions. A two-step RT-PCR method was used to determine relative mRNA quantities of various target genes. First-strand complementary DNA (cDNA) was synthesized from either 0.5 µg RNA in 50 µL RT reactions or 5.0 µg RNA in 100 µL RT reactions using the High Capacity cDNA RT Kit (ThermoFisher Scientific) following the manufacturer's instructions. TaqMan real-time PCR was performed using predesigned primers and probes with TaqMan Universal PCR Master Mix (ThermoFisher Scientific) in an Applied Biosystems StepOneplus Real-time PCR system to determine relative mRNA quantities of target genes with 18S ribosomal RNA as a normalizer. Serial dilutions of a representative cDNA sample were used to generate a standard curve for relative cDNA quantification. Relative mRNA quantities of target genes were normalized to the relative quantity of the 18S ribosomal RNA.

Statistical analysis

Statistical analyses were performed in GraphPad Prism 8.3 or higher (GraphPad Software, San Diego, CA). Scatter dot plots and error bars were presented as mean ± SEM. The Mann–Whitney or Kruskal-Wallis test followed by Dunn's multiple pairwise comparisons was used for

comparisons between 2 groups or ≥3 groups for data that were not normally distributed or did not have equivalence of variance. Otherwise, the unpaired or paired Student's t-test or one-way analysis of variance (ANOVA) followed by Tukey's or Dunnett's multiple pairwise comparisons test was used for 2-group or ≥3-group comparisons. For comparisons among groups over time or across lipoprotein fractions, two-way ANOVA with repeated measures and assumption of inequivalence of variance followed by Tukey's or Sidak's multiple pairwise comparisons test was used. The differences were considered statistically significant at $p \leq 0.05$.

Supplemental Figure S1

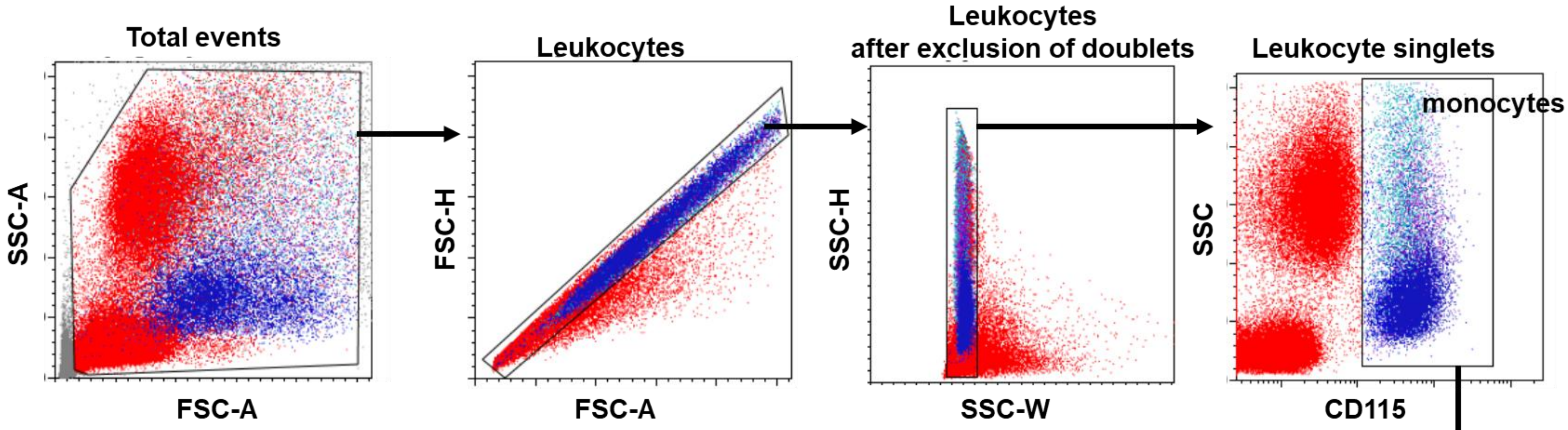

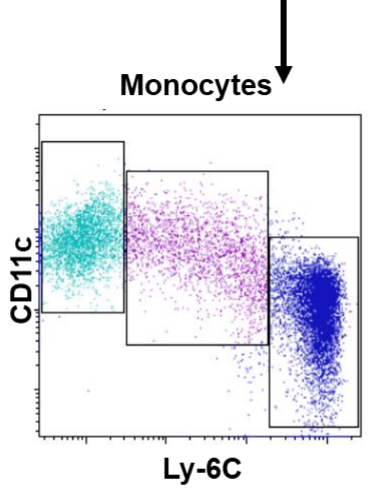

Supplemental Figure S1. Gating strategy for mouse monocytes.

A representative flow cytometry (FACS) example for monocyte gating strategy is presented. Blood was collected from an Ldlr$^{-/-}$ApoCIIItg mouse fed WD for 6 weeks, stained for CD115, CD11c, and Ly-6C, and analyzed for monocytes and subsets by FACS. Total leukocytes were first gated based on forward scatter area (FSC-A) vs side scatter area (SSC-A). Within the leukocyte gate, doublets were excluded based on FSC-A vs forward scatter height (FSC-H). Then leukocyte singlets were selected based on side scatter width (SSC-W) vs side scatter height (SSC-H). Total monocytes were defined as CD115^{+} cells within leukocyte singlets. Monocyte subsets were identified based on CD11c and Ly-6C within monocytes.

Supplemental Figure S2

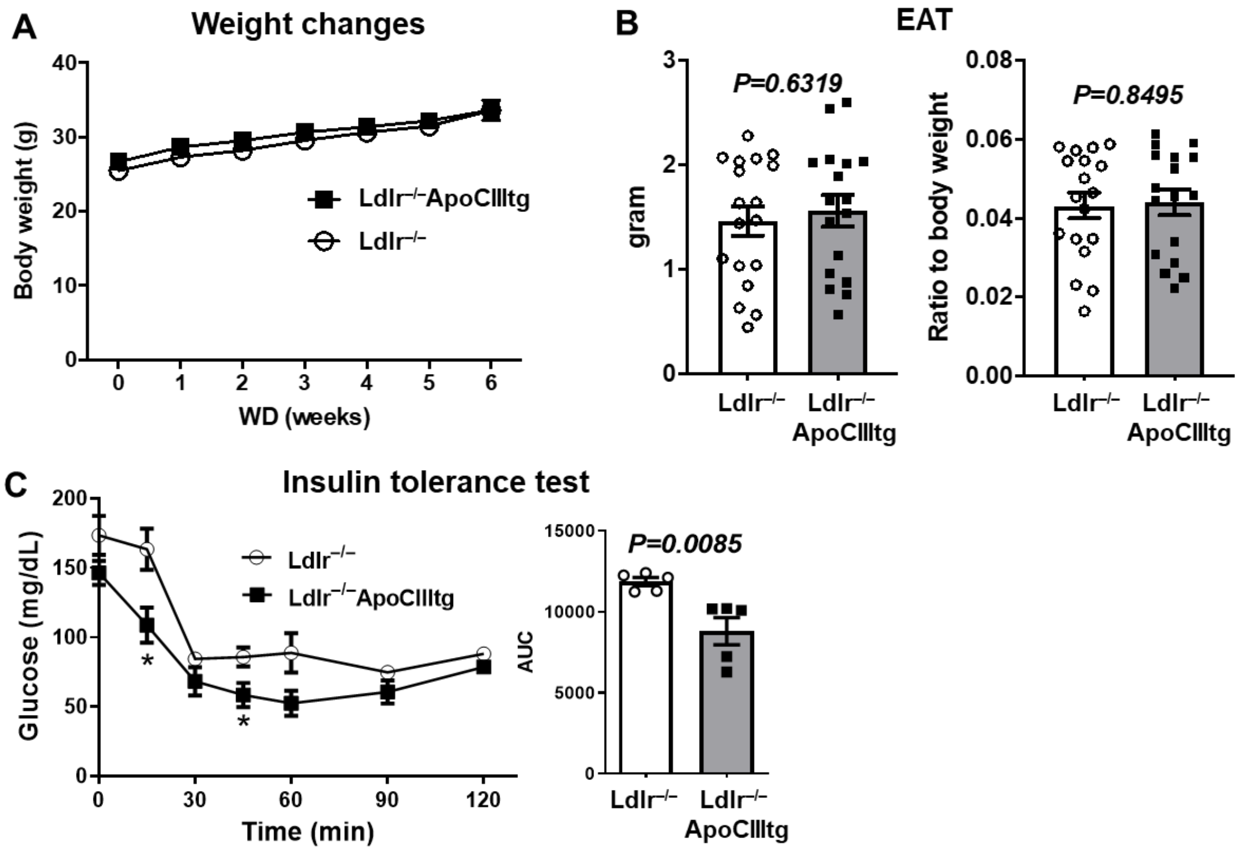

Supplemental Figure S2. Body weight changes, weight of epididymal adipose tissue (EAT), and insulin tolerance test.

Male Ldlr$^{-/-}$ApoCIIItg and Ldlr$^{-/-}$ mice were fed WD for 6 weeks. (A) Weekly changes in body weight. n=18–19 mice/group. (B) EAT weight (6-week WD). (C) Insulin tolerance test (6-week WD). Data are shown as mean ± SEM and were analyzed by two-way ANOVA with repeated measures and assumption of inequivalence of variance followed by Sídak‘s multiple pairwise comparisons test (A and C) or by unpaired Student’s t-test (B and C). *P <0.05 compared to Ldlr$^{-/-}$ group.

Supplemental Figure S3

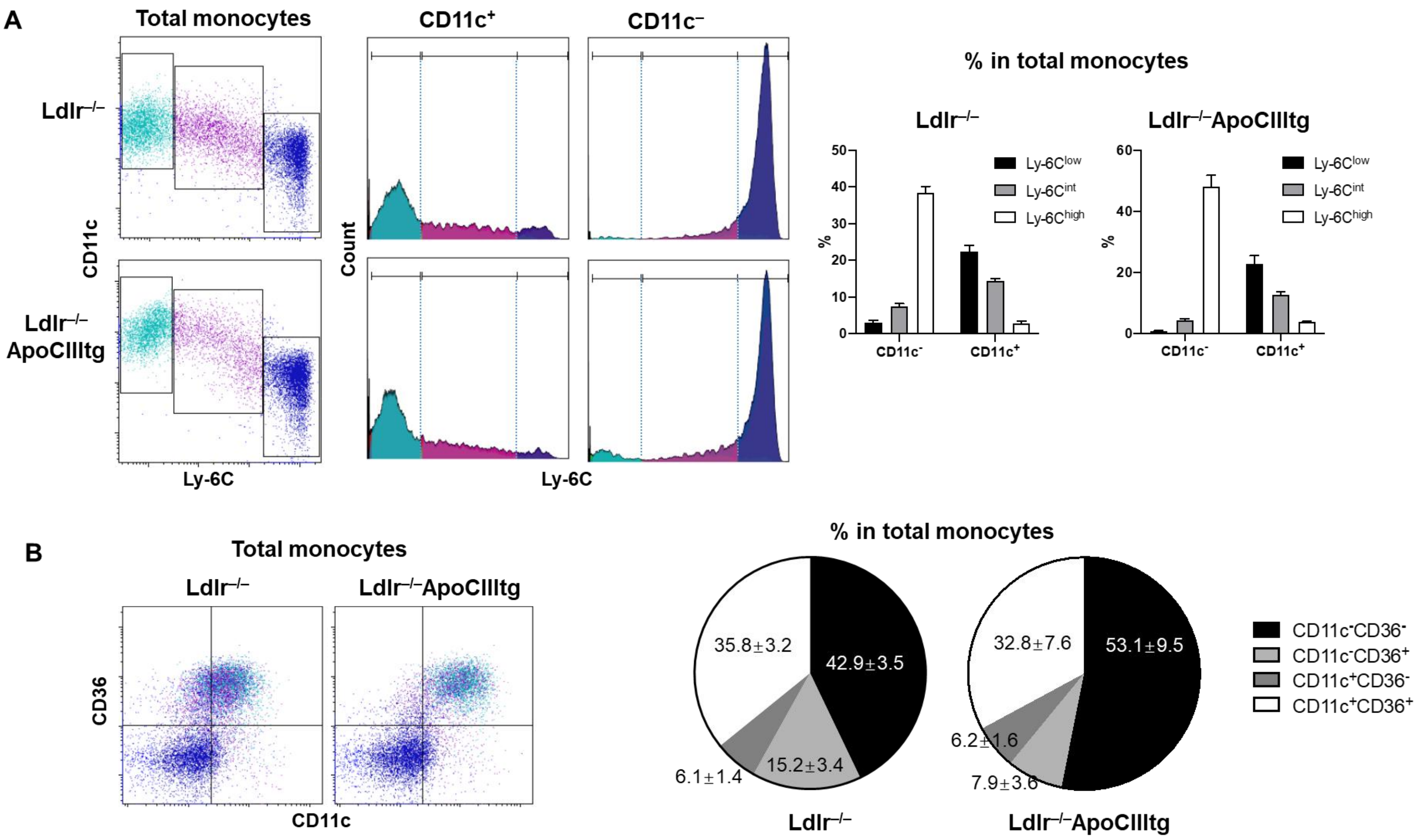

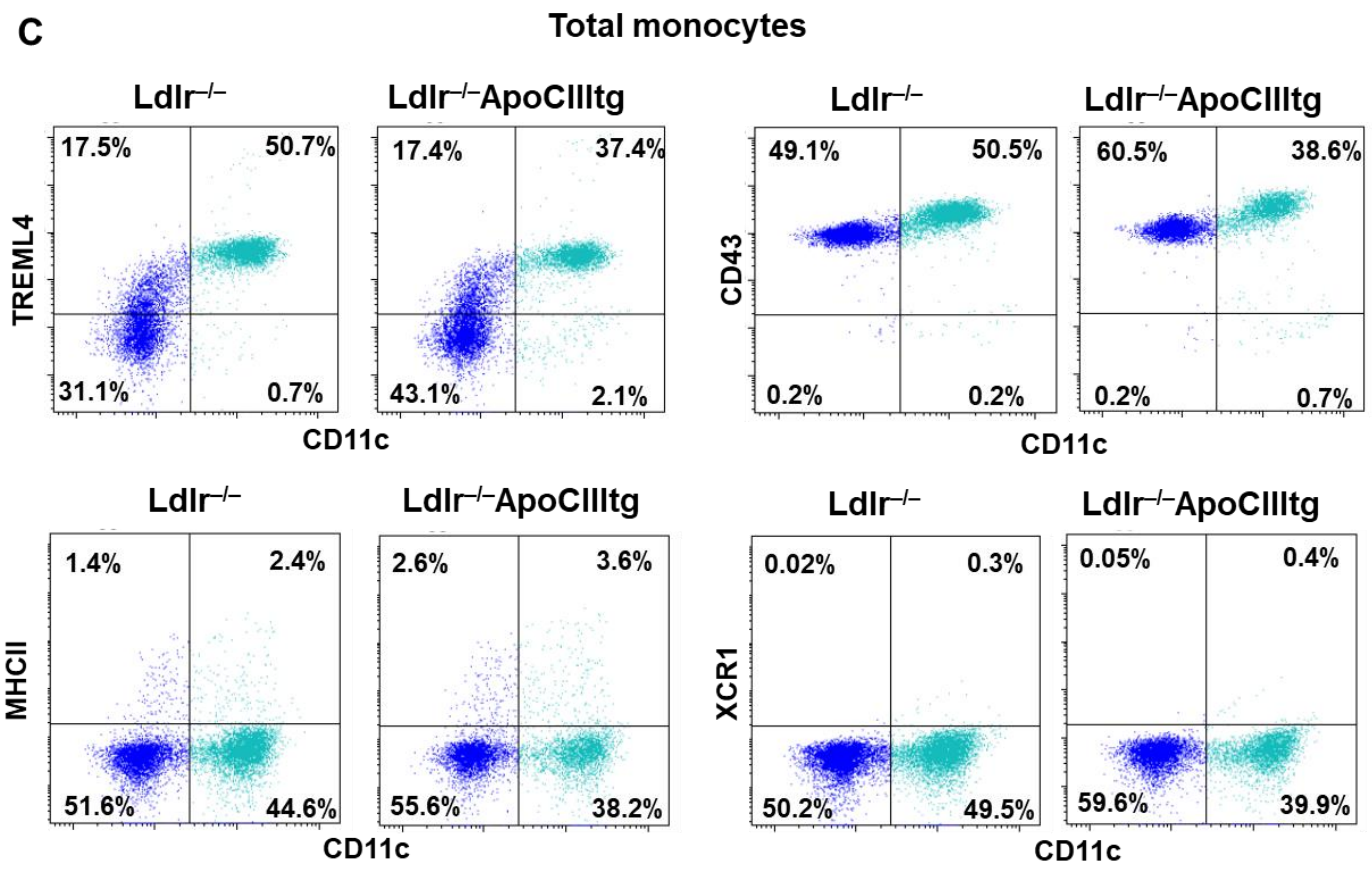

Supplemental Figure S3. Monocyte subsets in mouse blood.

Blood was collected from male Ldlr$^{-/-}$ApoCIIItg and Ldlr$^{-/-}$ mice fed WD for 6 weeks and analyzed for monocyte subsets by FACS. (A) Representative FACS examples and quantification of monocyte subsets based on CD11c and Ly-6C expression. (B) Representative FACS examples and quantification of monocyte subsets based on CD11c and CD36 expression. n=7 mice/group. Data are shown as mean ± SEM. (C) Representative FACS examples of expression of TREML4, CD43, MHCII, and XCR1 on monocyte subsets.

Supplemental Figure S4

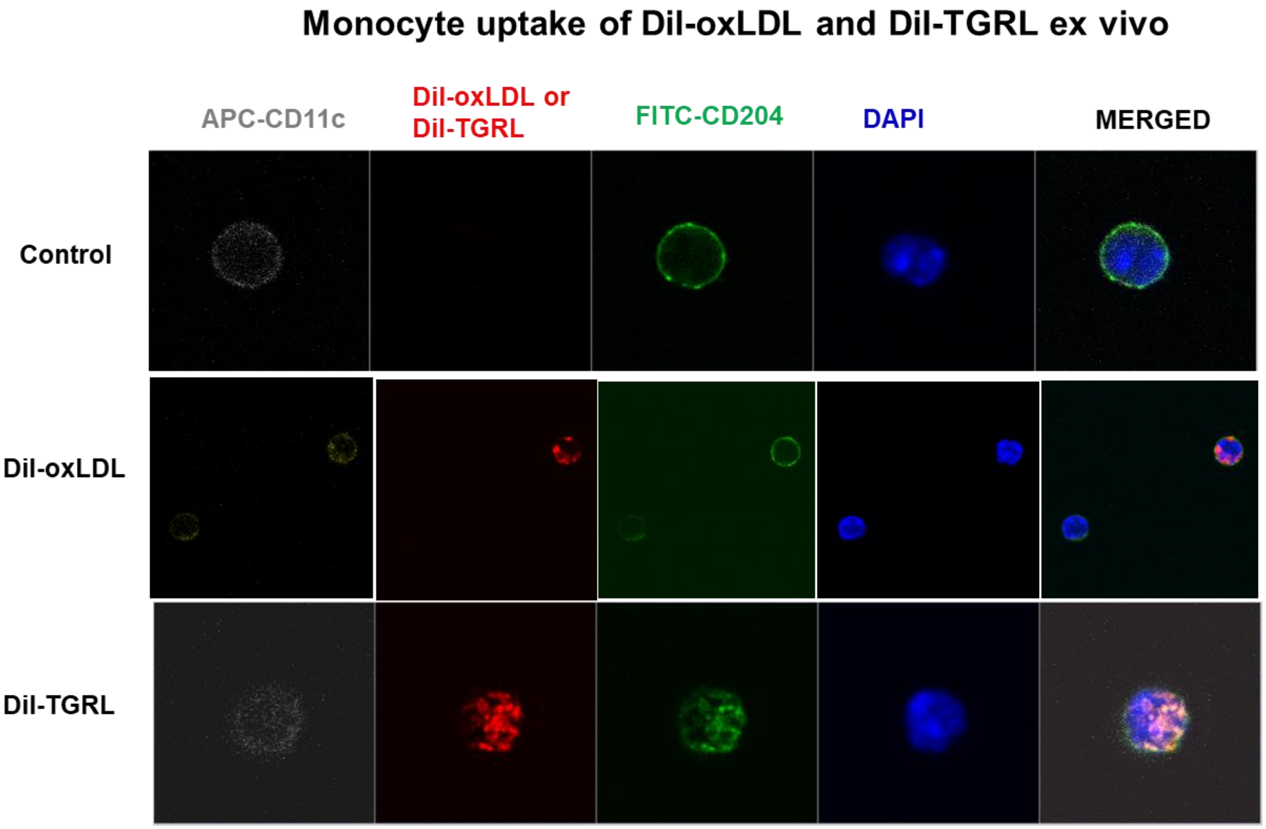

Supplemental Figure S4. Monocyte uptake of Dil-ox-LDL and Dil-TGRL ex vivo.

Representative images of monocytes incubated with Dil-oxLDL or Dil-TGRL showing monocyte uptake of oxLDL and TGRL. Blood from Ldlr$^{-/-}$ApoCIIItg (for oxLDL uptake) or WT (for TGRL uptake) mice was washed with PBS to remove endogenous lipoproteins and incubated with Dil-oxLDL or DiI-mTGRL in RPMI-1640 medium at 37°C for 3 hours. Then, red blood cells were lysed, and leukocytes were stained for CD204 and CD11c, fixed, and imaged by confocal microscopy. Control, no lipoproteins were added.

Supplemental Figure S5

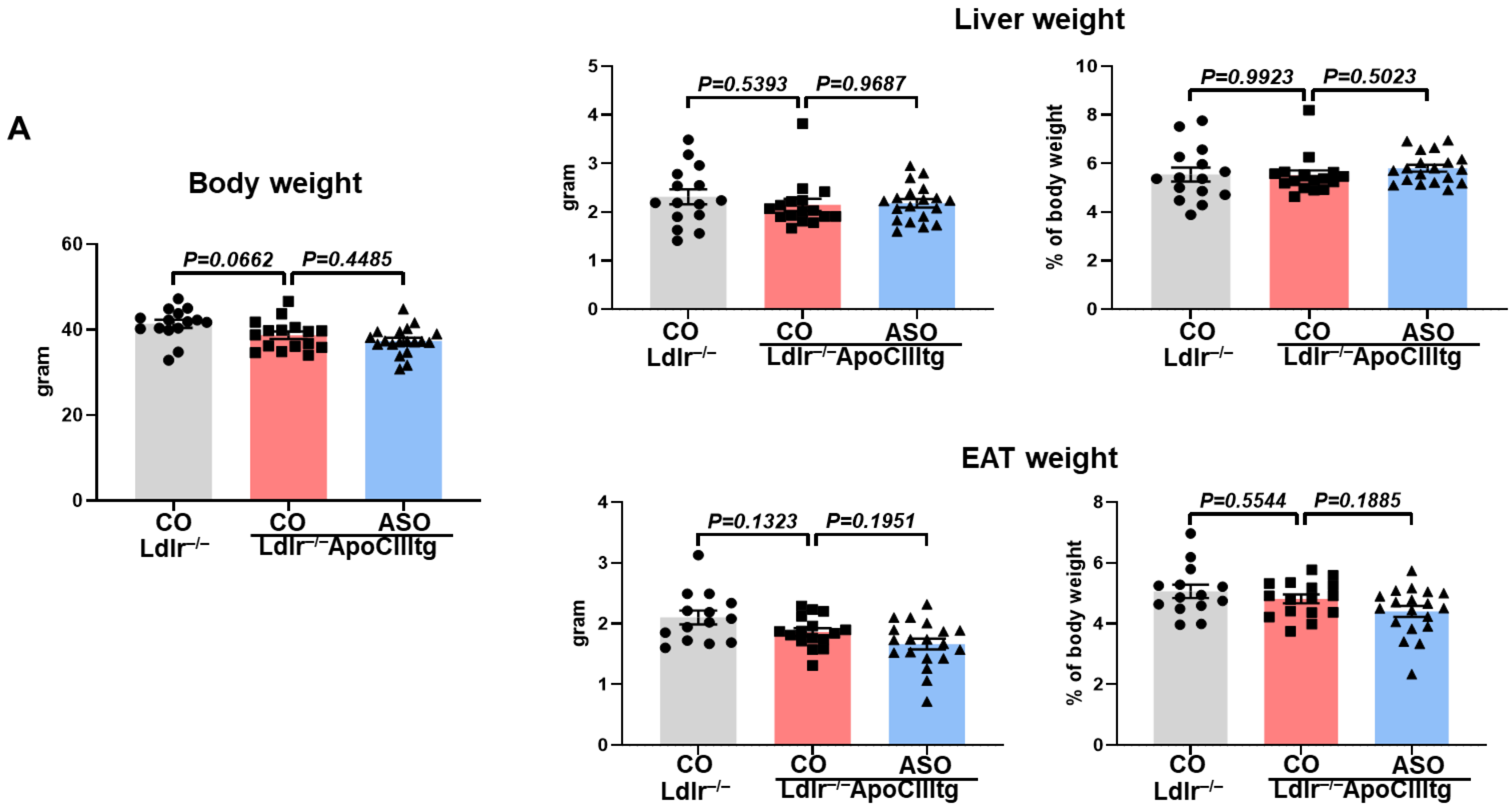

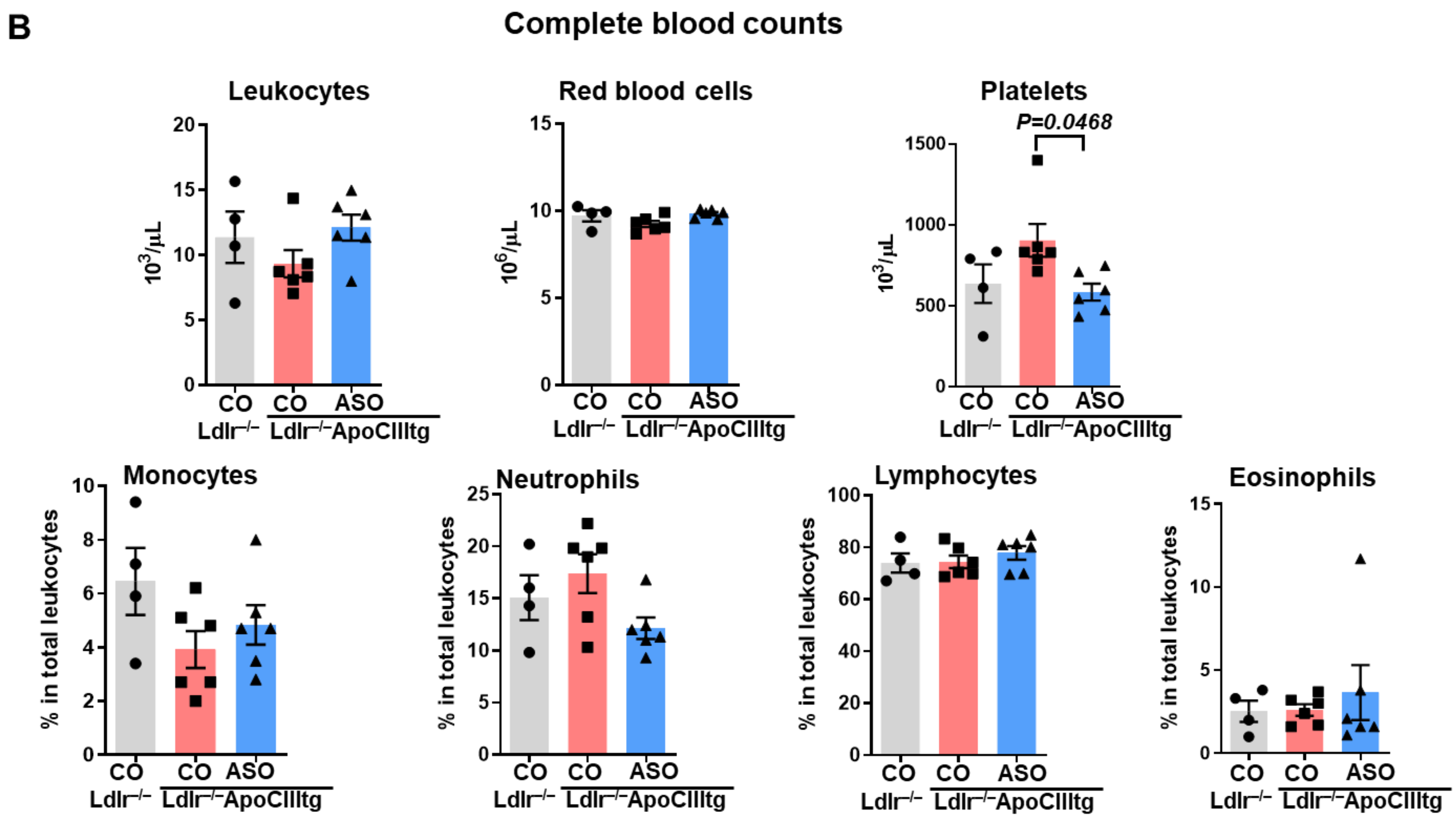

Supplemental Figure S5. Body weight and complete blood counts in Ldlr$^{-/-}$apoCIIItg mice treated with apoCIII ASO.

Male Ldlr$^{-/-}$apoCIIItg mice fed WD were treated with a GalNac-conjugated antisense oligonucleotide (ASO) against human apoCIII or a GalNac-conjugated control oligonucleotide (CO) weekly for 12 weeks. Male Ldlr$^{-/-}$ mice treated with CO were included as a control group. (A) Body weight and weight of liver and EAT at 12 weeks on treatment. n=15–19 mice/group. (B) Complete blood counts at 12 weeks on treatment. n=4–6 mice/group. Data are shown as mean ± SEM and were analyzed by one-way ANOVA followed by Tukey's multiple pairwise comparisons.

Supplemental Figure S6

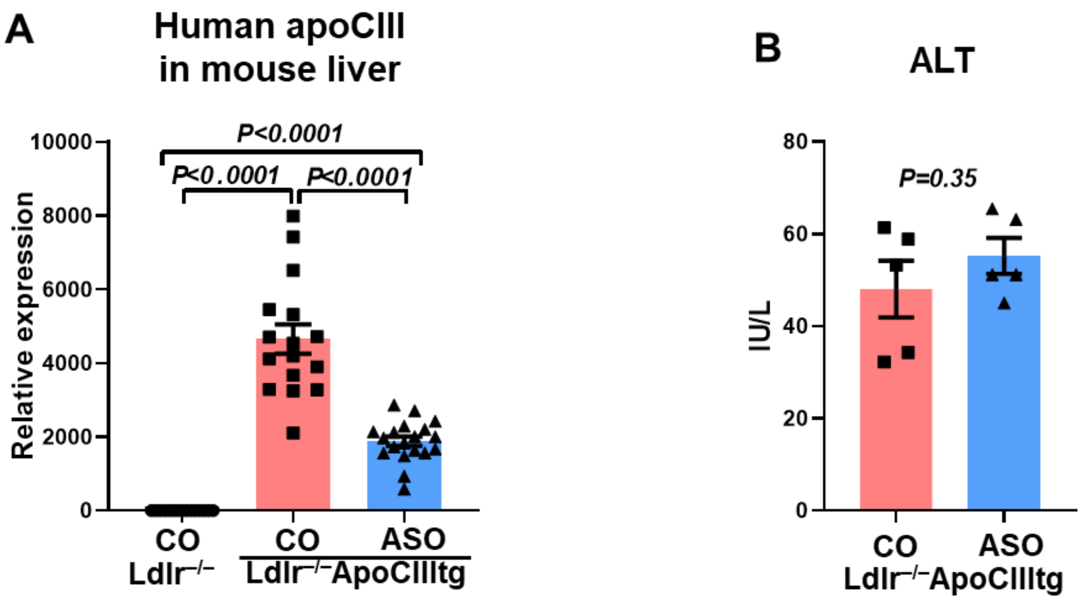

Supplemental Figure S6. Human apoCIII mRNA in the liver and alanine transaminase (ALT) levels in plasma of Ldlr$^{-/-}$ApoCIIItg mice treated with apoCIII ASO.

Male Ldlr$^{-/-}$ApoCIIItg mice fed WD were treated with ASO against human apoCIII or with CO weekly for 12 weeks. Male Ldlr$^{-/-}$ mice treated with CO were included as a control group. (A) mRNA levels of human apoCIII in mouse liver were examined by quantitative RT-PCR. n=15–19 mice/group. (B) Plasma ALT levels. n=5 mice/group. Data are shown as mean ± SEM and analyzed by one-way ANOVA followed by Tukey's multiple pairwise comparisons (A) or unpaired Student's t-test (B).

Supplemental Figure S7

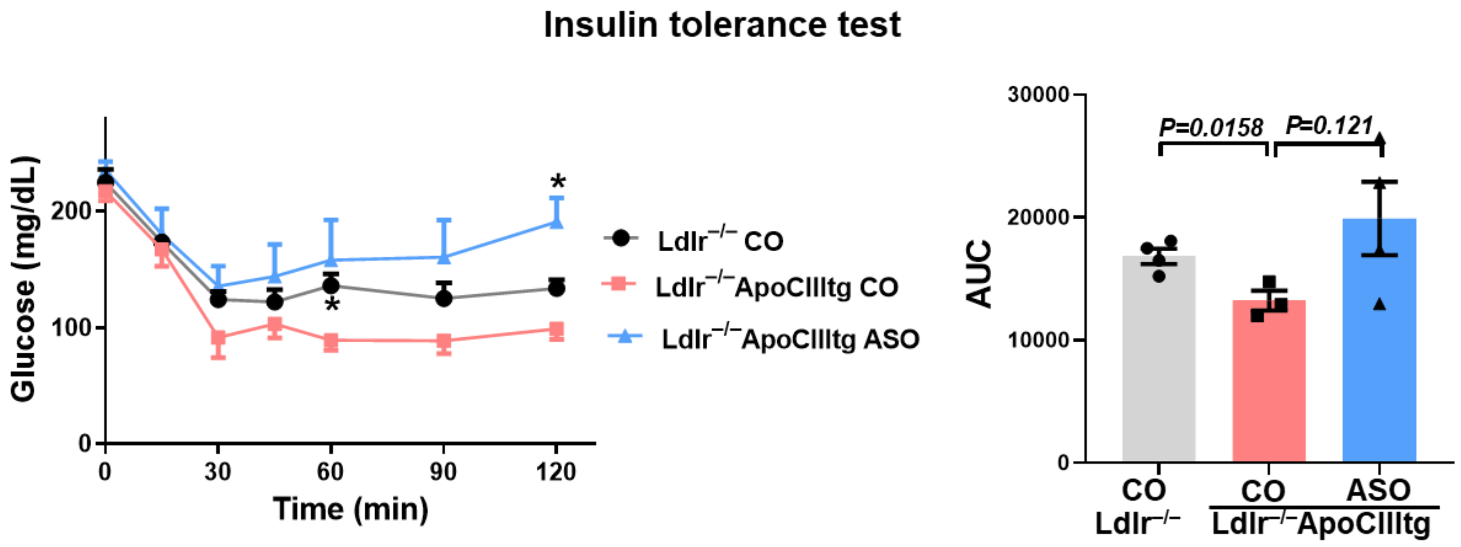

Supplemental Figure S7. Insulin tolerance test in Ldlr$^{-/-}$apoCIIItg mice treated with apoCIII ASO.

Male Ldlr$^{-/-}$ApoCIIItg mice fed WD were treated with ASO against human apoCIII or with CO weekly for 6 weeks. Male Ldlr$^{-/-}$ mice treated with CO were also included. Insulin tolerance test was performed after fasting for 6 hours. Data are shown as mean ± SEM and were analyzed using two-way ANOVA with repeated measures, assuming unequal variances, followed by Sídak‘s multiple pairwise comparisons test (for time-course data) or by unpaired Student’s t-test (for AUC data). *P <0.05 compared to Ldlr$^{-/-}$ApoCIIItg group.

Supplemental Figure S8

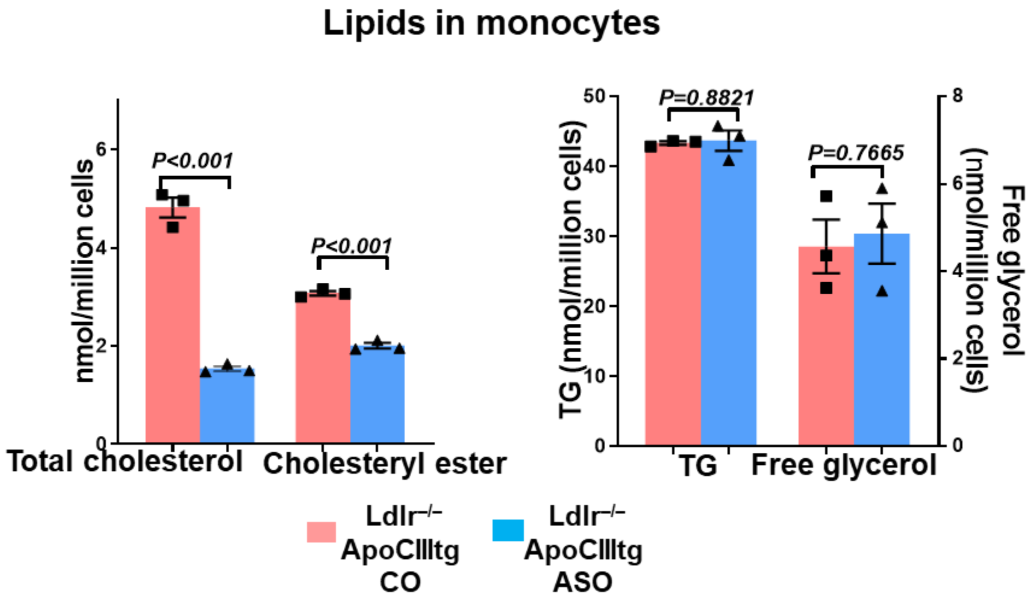

Supplemental Figure S8. Total cholesterol, cholesteryl ester, TG, and free glycerol in monocytes of Ldlr$^{-/-}$ ApoCIIItg mice treated with apoCIII ASO.

Male Ldlr$^{-/-}$ApoCIIItg mice fed WD were treated with ASO against human apoCIII or with CO weekly for 6 weeks. Monocytes were isolated from blood using RosetteSep™ DM-M followed by negative selection. Total cholesterol, cholesteryl ester, TG, and free glycerol levels were measured in monocyte lysates using the Triglyceride-Glo assay and Cholesterol/Cholesterol Ester-Glo assay kits and normalized to the cell number. n=3 mice/group. Data are shown as mean ± SEM and analyzed by unpaired Student's t test.

Supplemental Figure S9

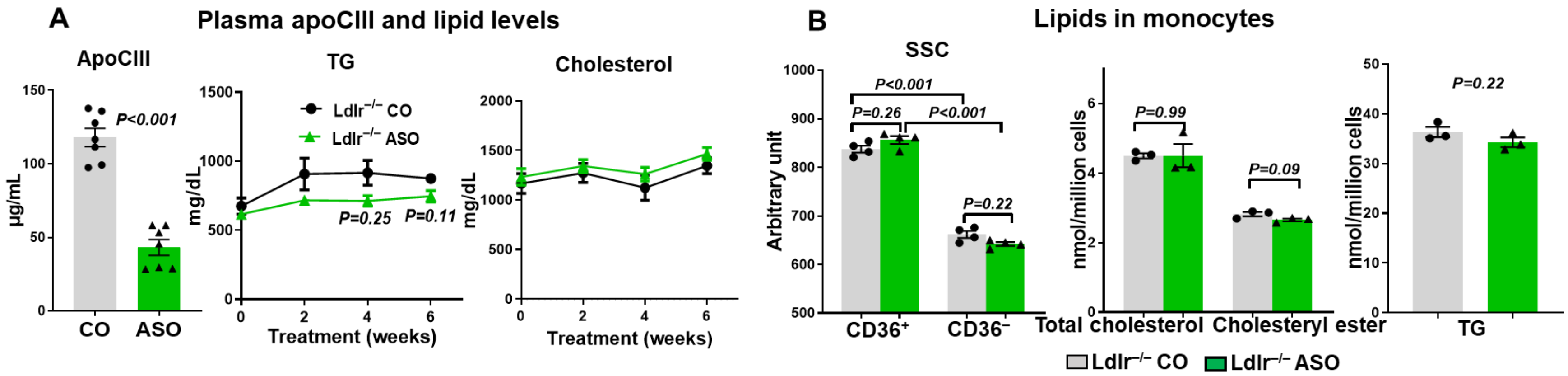

Supplemental Figure S9. Plasma apoCIII and lipid levels and monocyte lipids in Ldlr$^{-/-}$ mice treated with apoCIII ASO.

Male Ldlr$^{-/-}$ mice fed WD were treated with a GalNac-conjugated ASO against mouse apoCIII or a GalNac-conjugated CO weekly for 6 weeks. (A) Plasma levels of apoCIII, TG, and cholesterol. n=7 mice/group. (B) Monocyte lipids: Lipid accumulation in monocyte subsets indicated by SSC and cholesterol and TG content in monocytes measured in cell lysates and normalized to the cell number. n=3–4 mice/group. Data are shown as mean ± SEM and analyzed by two-way ANOVA with repeated measures and assumption of inequivalence of variance followed by Sídak‘s multiple pairwise comparisons test (A, TG and cholesterol), one-way ANOVA followed by Tukey’s multiple pairwise comparisons (B, SSC), or unpaired Student’s t test (A, apoCIII; B, total cholesterol, cholesteryl ester, and TG).

Supplemental References

1. Antony, A., Z. Lian, X. D. Perrard, J. Perrard, H. Liu, A. R. Cox, P. Saha, L. Hennighausen, S. M. Hartig, C. M. Ballantyne, and H. Wu. 2021. Deficiency of Stat1 in CD11c(+) Cells Alters Adipose Tissue Inflammation and Improves Metabolic Dysfunctions in Mice Fed a High-Fat Diet. *Diabetes* **70**: 720-732.

2. Khan, I. M., Y. Pokharel, R. T. Dadu, D. E. Lewis, R. C. Hoogeveen, H. Wu, and C. M. Ballantyne. 2016. Postprandial Monocyte Activation in Individuals With Metabolic Syndrome. *J Clin Endocrinol Metab* **101**: 4195-4204.

3. Lian, Z., X. D. Perrard, X. Peng, J. L. Raya, A. A. Hernandez, C. G. Johnson, W. R. Lagor, H. J. Pownall, R. C. Hoogeveen, S. I. Simon, F. M. Sacks, C. M. Ballantyne, and H. Wu. 2020. Replacing Saturated Fat With Unsaturated Fat in Western Diet Reduces Foamy Monocytes and Atherosclerosis in Male Ldlr(-/-) Mice. *Arterioscler Thromb Vasc Biol* **40**: 72-85.

4. Xu, L., X. Dai Perrard, J. L. Perrard, D. Yang, X. Xiao, B. B. Teng, S. I. Simon, C. M. Ballantyne, and H. Wu. 2015. Foamy Monocytes Form Early and Contribute to Nascent Atherosclerosis in Mice With Hypercholesterolemia. *Arterioscler Thromb Vasc Biol* **35**: 1787-1797.

5. Graham, M. J., R. G. Lee, T. A. Bell, 3rd, W. Fu, A. E. Mullick, V. J. Alexander, W. Singleton, N. Viney, R. Geary, J. Su, B. F. Baker, J. Burkey, S. T. Crooke, and R. M. Crooke. 2013. Antisense oligonucleotide inhibition of apolipoprotein C-III reduces plasma triglycerides in rodents, nonhuman primates, and humans. *Circ Res* **112**: 1479-1490.

6. Peng, X., Z. Lian, X. D. Perrard, Y. Xiao, J. Ni, V. O'Brien, H. Dong, H. J. Pownall, C. M. Ballantyne, and H. Wu. 2022. Poloxamer 407 Induces Hypertriglyceridemia but Decreases Atherosclerosis in Ldlr(-/-) Mice. *Cells* **11**.

7. Groeger, M., K. Matsuo, E. Heidary Arash, A. Pereira, D. Le Guillou, C. Pino, K. A. Telles-Silva, J. J. Maher, E. C. Hsiao, and H. Willenbring. 2023. Modeling and therapeutic targeting of inflammation-induced hepatic insulin resistance using human iPSC-derived hepatocytes and macrophages. *Nat Commun* **14**: 3902.

8. Wu, H., R. M. Gower, H. Wang, X. Y. Perrard, R. Ma, D. C. Bullard, A. R. Burns, A. Paul, C. W. Smith, S. I. Simon, and C. M. Ballantyne. 2009. Functional role of CD11c+ monocytes in atherogenesis associated with hypercholesterolemia. *Circulation* **119**: 2708-2717.

9. Daugherty, A., A. R. Tall, M. Daemen, E. Falk, E. A. Fisher, G. Garcia-Cardena, A. J. Lusis, A. P. Owens, 3rd, M. E. Rosenfeld, R. Virmani, T. American Heart Association Council on Arteriosclerosis, B. Vascular, and S. Council on Basic Cardiovascular. 2017. Recommendation on Design, Execution, and Reporting of Animal Atherosclerosis Studies: A Scientific Statement From the American Heart Association. *Circ Res* **121**: e53-e79.
